# Distilling a Visual Network of Retinitis Pigmentosa Gene-Protein Interactions to Uncover New Disease Candidates

**DOI:** 10.1371/journal.pone.0135307

**Published:** 2015-08-12

**Authors:** Daniel Boloc, Sergio Castillo-Lara, Gemma Marfany, Roser Gonzàlez-Duarte, Josep F. Abril

**Affiliations:** 1 Departament de Genètica, Facultat de Biologia, Universitat de Barcelona, Barcelona, Catalonia, Spain; 2 Institut de Biomedicina (IBUB), Universitat de Barcelona, Barcelona, Catalonia, Spain; 3 CIBERER, Instituto de Salud Carlos III, Barcelona, Catalonia, Spain; University of Florida, UNITED STATES

## Abstract

**Background:**

Retinitis pigmentosa (RP) is a highly heterogeneous genetic visual disorder with more than 70 known causative genes, some of them shared with other non-syndromic retinal dystrophies (e.g. Leber congenital amaurosis, LCA). The identification of RP genes has increased steadily during the last decade, and the 30% of the cases that still remain unassigned will soon decrease after the advent of exome/genome sequencing. A considerable amount of genetic and functional data on single RD genes and mutations has been gathered, but a comprehensive view of the RP genes and their interacting partners is still very fragmentary. This is the main gap that needs to be filled in order to understand how mutations relate to progressive blinding disorders and devise effective therapies.

**Methodology:**

We have built an RP-specific network (RPGeNet) by merging data from different sources: high-throughput data from BioGRID and STRING databases, manually curated data for interactions retrieved from iHOP, as well as interactions filtered out by syntactical parsing from up-to-date abstracts and full-text papers related to the RP research field. The paths emerging when known RP genes were used as baits over the whole interactome have been analysed, and the minimal number of connections among the RP genes and their close neighbors were distilled in order to simplify the search space.

**Conclusions:**

In contrast to the analysis of single isolated genes, finding the networks linking disease genes renders powerful etiopathological insights. We here provide an interactive interface, RPGeNet, for the molecular biologist to explore the network centered on the non-syndromic and syndromic RP and LCA causative genes. By integrating tissue-specific expression levels and phenotypic data on top of that network, a more comprehensive biological view will highlight key molecular players of retinal degeneration and unveil new RP disease candidates.

## Introduction

Retinitis pigmentosa (RP) is a highly heterogeneous genetic disorder with more than 70 known causative genes (see Retinal Information Network web site, RetNet [1]). It is the most common form of retinal dystrophies (RD), characterized by rod (responsible for dim light vision) and cone (daylight) photoreceptor cell degeneration resulting in night blindness and progressive vision loss [2]. Its prevalence is 1 in 4,000 individuals, following the Mendelian patterns of inheritance: autosomal-dominant (30–40% of cases), autosomal-recessive (50–60%) and X-linked (5–15%) [3]. Other reported inheritance patterns, such as digenic or mitochondrial, only account for a small portion of cases.

The identification of genes causing RP and closely related non-syndromic retinal dystrophies (such as Leber congenital amaurosis, LCA) has increased steadily during the last decade, yet around 30% of the cases remain unassigned. Therefore, present challenges in RDs are the identification of novel genes, the understanding of the main cellular processes altered in this group of pathologies, and the design of effective therapies to halt, ameliorate or cure the disease [3].

Although a considerable amount of genetic and functional data on single RD genes and mutations has been gathered, a comprehensive view is still missing to navigate through gene functions, cellular pathways and pathogenicity. In contrast to the analysis of single isolated genes, finding the networks that link disease genes provides powerful etiopathological insights. Indeed, molecular medicine based on gene and protein networks has been rapidly expanding and has shown that most disease-causing genes often work together, either forming a protein complex or participating in the same signaling pathways, clearly underscoring the connection between disease and gene/protein networks [4].

Network-based approaches are becoming increasingly valuable to analyze complex systems of interaction, and also to explore high dimensional data. The whole functional and regulatory cell network is made up by a myriad of molecular interactions, either physical—where direct associations between molecules constitute protein complexes, signaling and metabolic pathways—, or genetic—where regulation of gene expression, mediated by transcription factors and interfering RNAs, translates into phenotype—[5]. This strategy has already been instrumental in the identification of causative genes in breast cancer, schizophrenia, and cerebral ataxias, among others [6–12]. Complex behaviors emerge with the visualization of network interactions among the proteins/genes involved in a specific disease, and project beyond direct physical interactions, since these molecular components play multiple roles in a pathway, regulate transcription and splicing processes, and even contribute to larger functional complexes without actually physically contacting each other. Finally, networks could also be useful to elucidate new candidates, highlighting genes that have connections to known disease genes, or linking two or more disease genes (acting as a bridge).

The advent of exome and genome sequencing has provided an extremely useful tool for routine genetic diagnosis and novel gene search for highly heterogenous hereditary disorders, such as RP. However, prioritization of previously reported and new candidates after identification of exome-based genetic variants, and assessment of their pathogenicity remain a bottleneck in molecular diagnosis. Indeed, to provide a more comprehensive view of the molecular and cellular basis of the disease, which is indispensable to approach efficient therapy, we need new tools and paradigms. This type of approach has already been explored by geneticists and network researchers, but the networks generated are usually not searchable[11,13]. A web application, such as RPGeNet, may become a useful tool for research in rare diseases, similarly to the interactome databases for disease currently being implemented in the world of cancer research [14–16]. Although current available gene-centric tools allow to navigate through interactions, such as GeneMANIA [17,18], or facilitate the reconstruction of disease subnetworks from expression profiles, like EgoNet [6], in the field of retinal diseases there is no such a tool to visualize specific gene or protein interactions among RP/LCA causative genes. Consequently, information has to be gathered from many complex databases, a burdensome and time-consuming task for most researchers. In this context, one of our aims was to develop a user-friendly searchable web application, RPGeNet [19], that integrates all the physical and genetic interactions obtained from different databases, as well as other relevant genetic and functional data, i.e. tissue-specific expression levels and the frequency of genetic variants per gene. This tool is specifically focused on the interactions subnetwork for the RP/LCA genes.

## Results and Discussion

**Fig 1:**
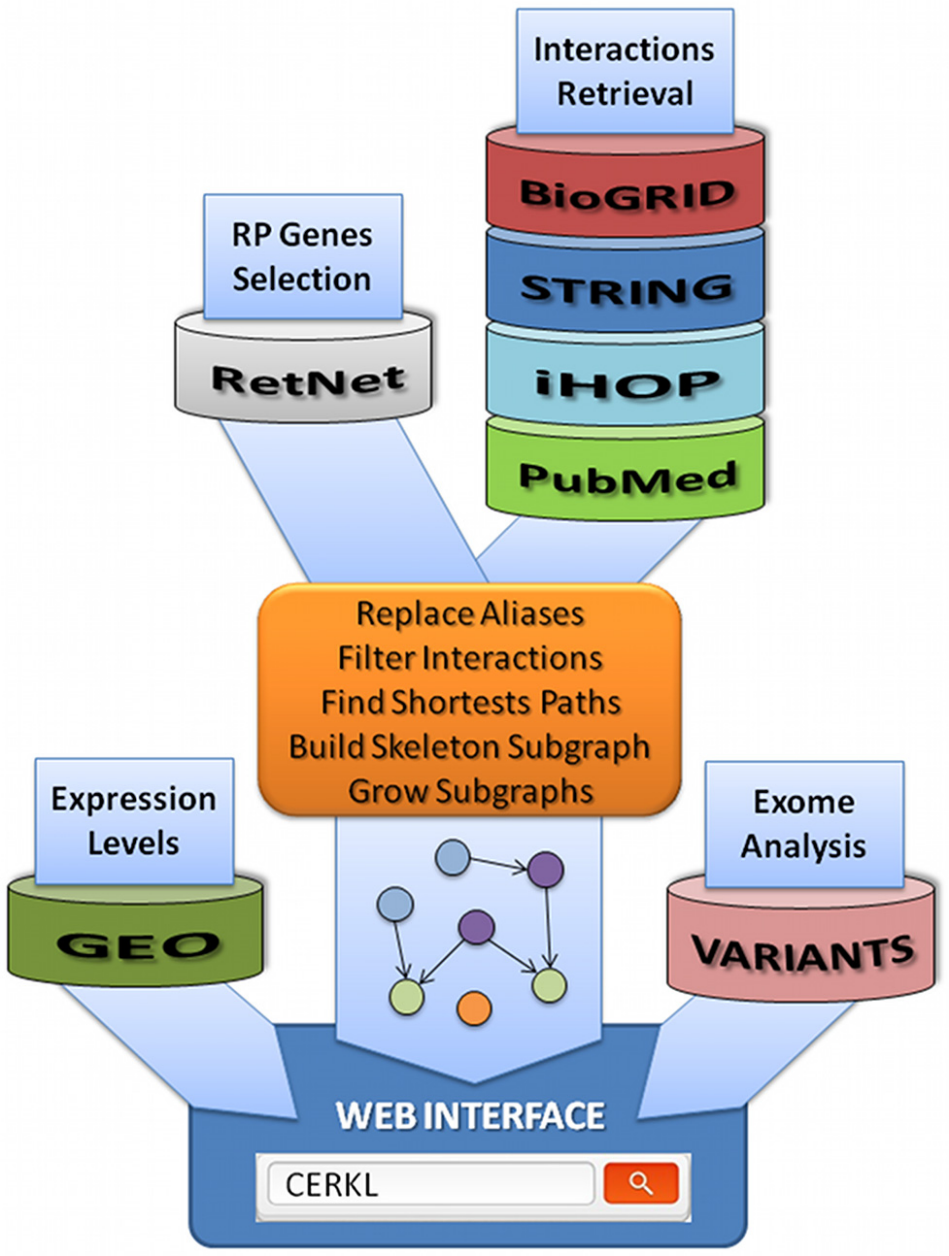
Schema of the protocol used to derive the RP/LCA genes network. Driver (RP/LCA) genes were selected from RetNet, while interactions were gathered from a variety of sources. Main script combines such information to build the driver subnetwork that is the kernel of the web interface. Further information, such as gene expression or number of reported polymorphisms, is integrated when navigating through the selected paths.

We expected to identify shared molecular functions and mechanisms, indicating new potential causative candidates and disease-modifying genes as well as novel targets for efficient therapies. Our approach relied on the connectivity of the resulting networks generated by integrating: 1) protein-protein (BioGRID [20,21], STRING [22]) and gene-gene (STRING) high-throughput (HT) data; 2) manually curated data from the Information Hyperlinked over Proteins (iHOP) repository [23], which facilitates syntactical searches by keyword for interactions from the scientific literature; and finally, 3) relevant related published information, available from PubMed [24], on protein/gene interactions specific for non-syndromic RP and LCA plus a selection of syndromic RP/LCA genes. The list of the 110 genes used as baits in this analysis is provided in the S1 Table (selected from RetNet, details in the Methods section). Once all the interactions were selected, we used a Perl script to generate an initial skeleton (level 0) of interactions from which the network at different distances was grown by adding up neighborhood nodes. Indeed, when more levels were added to the network, it became very noisy and overpopulated, hampering the visualization of connections or patterns that were evident at lower levels. Fig 1 provides a general overview for the whole process.

When building the network, we focused on an integrated approach where the total interactions retrieved from the aforementioned sources were used as the global RP/LCA database (summarized in column “ALL DBs” in S2 Table). Overall, after screening the collected interactions spanned to as many as 63,139 nodes (genes and proteins) and 1,688,656 edges (interactions). Within the whole set of nodes, we were able to detect as much as 103 RP genes. The genes *C2orf71*, *DTHD1*, *HGSNAT*, *IMPG2*, *KCNJ13*, *PRCD*, and *SLC7A14*, most of them identified recently, remain unconnected and clearly demand further functional studies to integrate them in an interaction network.

**Fig 2:**
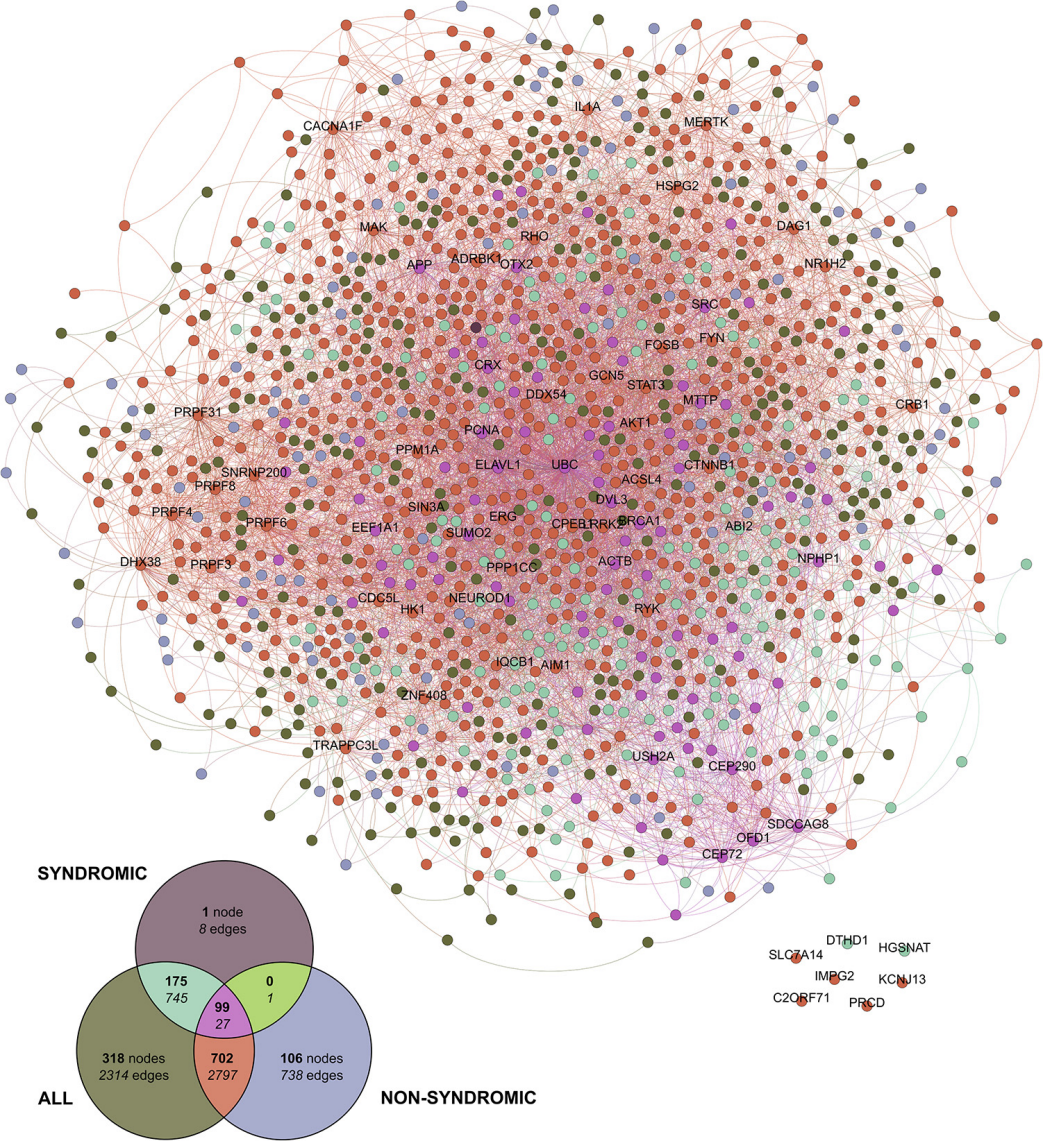
Skeleton graph for the shortest paths between pairs of RP/LCA genes. RP/LCA genes were split into three sets: those annotated as non-syndromic (75 genes), those as syndromic (35 genes), and a third set containing both (110 genes). A skeleton graph based on the shortest paths was built for each of those three sets. The resulting graphs for the two small sets were projected over the complete one; this intersection provides clues on the nodes and interactions captured using each driver genes sets. Left bottom Venn diagram summarizes the number of nodes (in bold face) and interactions (in italics). At the same time, it provides a color legend to explain the source of the nodes and interactions drawn on the main graph plot. Labels of the highly connected nodes (those having more than 40 incoming or outgoing edges), as well as for the 7 driver genes that remain unconnected, show the corresponding HUGO symbol identifier. It is worth to mention that dark green nodes are mainly defined by genes/proteins providing new paths to connect non-syndromic and syndromic genes.

One of the main efforts was to produce a core set of interactions, using the shortest path connecting every pair of RP genes. The second section of S2 Table summarizes the graph properties of the skeleton network. The resulting graph for the skeleton RP subnetwork has 1,287 nodes and 5,883 edges (see Fig 2). On top of the skeleton graph, for the whole network we have projected the skeletons built from the two subsets of RP/LCA genes, non-syndromic and syndromic, using a color code, which is synchronized with the Venn diagram at the bottom left. We provide the gene symbols of the highly connected RP/LCA skeleton nodes (more than 40 connections), and the 7 genes that remain unconnected (nodes at the bottom right).

The high connectivity degree hampers the visualization of particular interactions, even though the graph has a low density value and merely represents 7.8 per thousand of all possible connections. This distilled skeleton graph was used as the basic aggregating network that was progressively expanded into a multilevel approach following a “parent-to-child” (aka “source to target”) interaction bidirectional growth, from levels 1 to 4. The growth of the recovered interactions for each RP gene per level is illustrated in S3 Table. A saturation of the retrieved interactions between nodes is reached at level 1 for most of the RP genes, which is consistent with the protocol applied as the parent and child nodes outside the shortest paths between RP/LCA genes are added on the first iteration over the skeleton graph. Notably, the average degree for the whole set of nodes on the skeleton and level 1 graphs is 9.14 and 33.0 respectively, while the average degree of the RP/LCA genes at each level ranges from 22.1 up to 59.6, converging to that value at level 2 iteration (see bottom rows of S3 Table). Considering this analysis of the network expansion, we estimate that level 1 provides a reasonable core network to link RP/LCA causative genes with other putative candidates involved in the disease. Indeed, the size of the core network has a direct impact on the usability of the interactive visualization, even when producing static views of the net. As it is widely accepted in the field, the connectivity degree of a given node does not need to reflect its real biological relevance in the whole network, although those nodes that are important for a given function can be often detected as sub-network hubs [16,25]. However, connectivity can be also biased towards the “popularity” of the gene/protein defining a node, as the interest of the scientific community in a specific gene could be not directly related to its molecular role [26]. Therefore, for the webapp visualization of the network we have not relied on connectivity to weight the node size, and instead relied on other biological features, such as number of genetic variants (reported polymorphisms and mutations, as detailed in the Methods section). Finally, it has been described that higher average clustering coefficient is correlated with a high level of redundancy and cohesiveness [27], which is reflected in the corresponding rows of S2 Table when comparing the aforementioned variable on skeleton versus level 1 graph.

Before merging the data, we have assessed the specific contribution of each dataset to the connectivity of the core net; for this purpose, a preliminary set of 62 non-syndromic RP genes was used. Two complementary approaches were considered: interactions derived from syntactic analysis of the literature and data produced by high-throughput means. Literature-curated data usually have a biological context, which supports the physiological relevance of the retrieved interactions, but it is clearly biased towards well-known genes and proteins. In contrast, Omics data is unbiased but lacks context, which hampers the assignment of functional roles. This is reflected in S2 Table, when comparing the summary columns of Sparser (Stanford-parser syntactic analysis) and iHOP (pre-computed syntactic analysis) with those of BioGRID and STRING (HT repositories). Concerning syntactic analyses, we complemented the manually curated data retrieved from iHOP (a well-established interaction database) by performing a custom automated syntactic analysis (Sparser) on electronic Abstracts and Full Texts from articles recovered specifically after a general search for “*retinitis pigmentosa*”. This combined approach has provided valuable data, recovering 28 new interactions and connecting 11 additional nodes in the skeleton graph, since there has been an explosion of articles describing interactions for the RP genes in the last five years, which were not well represented in our manually-curated set derived from iHOP (illustrated in the S1 Fig upper panel histogram, as well as in the small overlap between the two sets shown in S2 Fig panel 3 Venn diagram).

**Fig 3:**
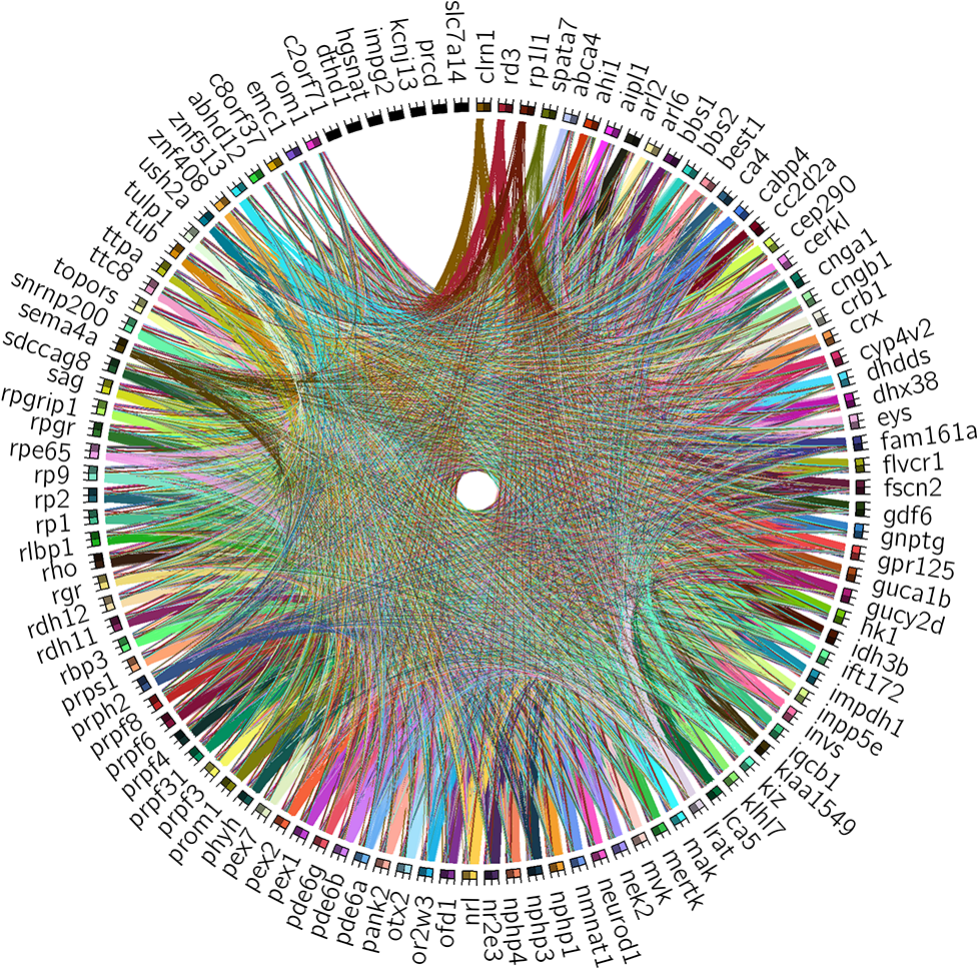
Summary of the RP/LCA genes pair-wise connectivity. On the outer ring of the diagram we have depicted the standard symbols for the 110 RP/LCA genes used to build the network, sorted first by the number of incoming, then by outgoing connections, and finally, by alphabetical order. Each driver gene that interacted with another RP/LCA gene is shown by a distinct color box, while those for which there was no reported interaction are shown in black. The colored boxes are divided in two moieties: the lighter half groups all the outgoing connections—thus reflecting a directed path starting from the current RP node—, whereas the darker half gathers all the incoming connections—indicating a directed path ending at the current RP node. This differentiation in two halves facilitates to spot genes upstream (e.g. *CLRN1* and *RD3)* or downstream *(e*.*g*. *ROM1* and *EMC1)* of many others in the network, or even internal hubs (e.g. *CERKL*, *CRX*, and *RP2*). This figure was obtained using Circos [28]. Further details and pairwise connectivity growth for all, non-syndromic and syndromic gene sets are available in S3 Fig.

When considering all the interactions among the RP/LCA genes from the skeleton graph, the number of intermediate nodes can be calculated to provide a distance value between each pair of nodes. A diagram summarizing incoming and outgoing connections for all genes and its growth from distance 1 to 6 is shown in Fig 3. The genes with only outgoing or incoming connections group with those unconnected. Remarkably, the functions of all these genes is mostly unknown. On the other hand, genes encoding splicing factors (e.g. PRPF factors), enzymes (e.g. subunits of the PDE6, kinases such as MAK, MERTK, MVK) and ciliary proteins (NPHP proteins) show an equilibrated incoming and outgoing connectivity. It is worth mentioning that when dissecting the interactions by measuring the pairwise distance (see S3A Fig), proteins involved in functional high molecular complexes (spliceosome factors, retinal specific transcription factors, phototransduction) or localized in specialized subcellular compartments (peroxisome, ciliary body…) become apparent at distance 1. This effect is mostly contributed by non-syndromic genes (as can be noticed when comparing plots from S3B Fig and S3C Fig). As a result of the high connectivity degree already mentioned–S3 Table, average incoming degree of 9.87 and outgoing degree of 12.22–we observe most of the pairwise shortest path connectivity for RP/LCA driver genes at distance 3. The biological explanation is that an average of 2 intermediate non-RP nodes are required to link RP/LCA genes, which provide future candidates to focus on functional studies as well as in genetic analyses. Notably, the connectivity of syndromic genes saturates at level 4 (S3C Fig), indicating a higher density of connections at lower levels and supporting the pleiotropic effects produced depending on the gene mutation.

**Fig 4:**
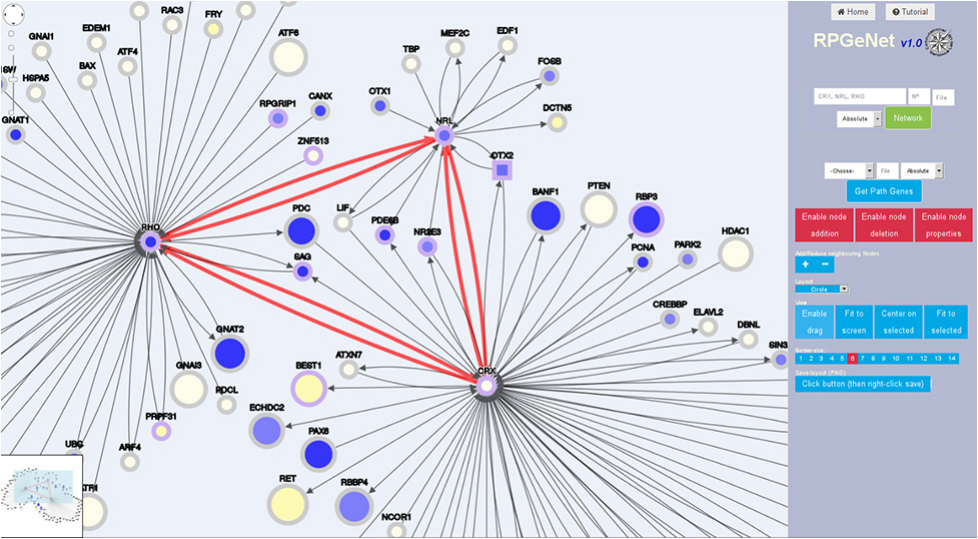
RPGeNet visualization focusing on CRX, NRL and RHO partners. Snapshot of the customized network of interaction partners retrieved from RPGeNet when querying for *CRX*, *NRL* and *RHO* genes. This node distribution was derived from a basic circular layout, manually rearranged on the webapp by the researcher to underscore the relationships among those three genes. Neighbor nodes at distance 1 were requested upon the basic skeleton graph (level 0). The RP/LCA genes are highlighted by a light violet border in contrast to the grey colored non-RP genes, square nodes indicate syndromic RP/LCA genes. The node size is proportional to the number of variants in coding regions (neutral or mutagenic). The core color reflects the relative expression level in retina with respect to the average in all tissues, as calculated from the GEO GSE7905 dataset. The shades range from blue (over-expressed in retina) to yellow (under-expressed). Edges connecting the query RP/LCA genes were drawn in red, just taking advantage of the edge selection feature available from the webapp.

RPGeNet is built upon those distilled sets of interaction at distinct levels. The usefulness of this webapp from the molecular standpoint is illustrated by Fig 4, which shows an snapshot of the interaction networks centered on three well known RP genes, namely the transcription factors *CRX* and *NRL* and one of their target genes, *RHO*, neighbouring nodes at distance 1 on the default level (0—skeleton graph). This basic query using this trio of RP genes–the red links highlight their interactions–resulted in an expanded network including nine additional RP/LCA related genes, *BEST1*, *NR2E3*, *PDE6B*, *PRPF31*, *RBP3*, *RPGRIP1*, *SAG*, *ZNF513* and the RP syndromic *OTX2* (depicted as a square node). This comprehensive approach gives very valuable information in a single picture, spotting some of the genes encoding known transcription factor protein complexes, such as *CRX*, *NRL*, *NR2E3*, *OTX2*, *OTX1*, *CREBBP*, and adding some chromatin remodeling partners, e.g. *HDCA1*, *BANF1*… which could play a role in the regulation of relevant retinal genes. Another illustrative example is the unveiling that two other relevant genes causing distinct neurodegenerative disorders a priori unrelated to the retina, *ATXN7* and *PARK2*, interact with *CRX*. *ATXN7* was initially described as one of the main factors causing human ataxia, but has recently been involved in macular degeneration after being revealed by interaction network analysis [11]. On the other hand, *PARK2* is one of the causative genes of Parkinson’s Disease, which encodes a ubiquitin E3 ligase involved in autophagy. The formation of autophagosomes is extremely relevant for the retinal homeostasis, and *PARK2* protects the retina from light-induced degeneration.

**Fig 5:**
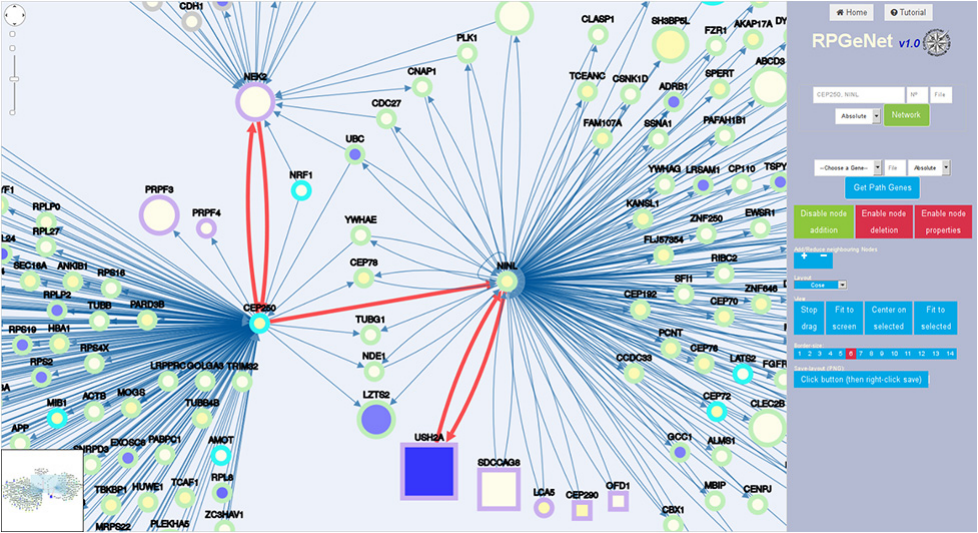
RPGeNet-mediated discovery of putative RP/LCA-genes pathways. Snapshot of the RPGeNet generated graph, where the nodes have been reordered to show the interactions, captured when searching for nodes at distance 1 of *CEP250* upon the level 1 subnetwork. This query using a non RP-gene retrieved a sub-network that includes 8 RP/LCA-genes (four of which are non-syndromic). As described in the Results section, *CEP250* conforms a specific pathway relating *NEK2* (RP gene) to *USH2A* (a gene causing RP and Usher syndrome), via *NINL* (edges highlighted in red). These bridging genes are all good candidates to contribute to retinal dystrophies. The RP/LCA genes are highlighted by a violet border, while border color for “parent” and “child” nodes is indicated by light blue and green, respectively. The shape, border and core color, as well as node size, are defined as in Fig 4.

Direct queries using genes that have not been reported to cause RP can also be performed to search for specific new disease candidates. Fig 5 illustrates the network generated after querying for *CEP250*, selecting neighboring nodes 2 on the level 1 subnetwork (built by adding all the nodes that are at distance one from those at the skeleton graph). Notably, up to eight RP/LCA genes appear in the network centered on this gene. Particularly, it is located in the linking path between *NEK2* to *USH2A*, *SDCCAG8*, *LCA5*, *CEP290*, and *OFD1*, highlighting its relevance in retinal molecular pathways. In this context, the late report combining mutations in *CEP250* with *C2orf71* associated to severe deafness and blindness (Usher syndrome) emphasizes its probable role as a RP causative or a disease-modifying gene [29]. Indeed, this figure also emphasizes the hub status of *CEP250* next neighbor, *NINL*, which not only interacts with many proteins but links Usher syndrome and Leber congenital amaurosis (two syndromic severe retinal disorders) in agreement with being an excellent candidate for causing ciliopathies and other retinal disorders [30].

**Fig 6:**
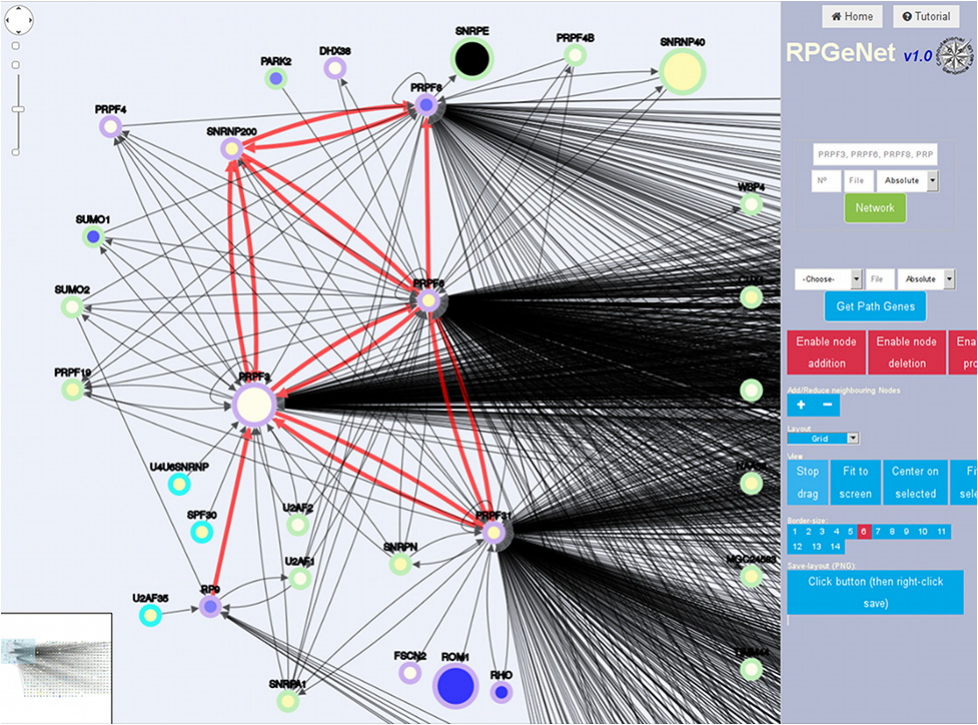
Network of RP/LCA genes involved in splicing and their partners. RPGeNet snapshot of the edited graph derived from the original grid layout (ordered by connectivity of the nodes, from left to right, and top to bottom) when the query was performed with the RP genes involved in the splicing machinery, namely *PRPF3*, *PRPF6*, *PRPF8*, *PRPF31*, *SNRP200*, and *RP9*. In this case, neighbor nodes at distance one were requested upon the level 1 graph. The shape, border and core color, and node size are defined as in Fig 5. Edges connecting the query RP genes were drawn in red. Note that five additional RP/LCA genes were also retrieved by this query: *RHO*, *ROM1*, *FCSN2*, *DHDX38* and *PFPF4*.

Fig 6 specifically focuses on the RP genes involved in splicing and their closest interactors, namely, the query included *PRPF3*, *PRPF6*, *PRPF8*, *PRPF31*, *SNRP200* and *RP9*, selecting neighboring nodes at distance 1, over the graph at level 1. Again, the red links highlight the interactions among the queries. The PRPF proteins are tightly connected among them and highly connected to other target nodes. Notably, and in comparison with the previous example (Fig 5), only five additional RP genes, *RHO*, *ROM1*, *FCSN2*, *DHDX38* and *PFPF4* (also a component of the spliceosome) appear in this network, which is in accordance to their relevant role in a ubiquitous basal cellular process (splicing) that affects many target genes, not exclusive of the retina. The ubiquitous role of the splicing machinery proteins may be reconciled to the unique affectation in the retina when considering the high transcriptional diversity of retinal genes due to the complexity of alternative splicing, as revealed after high-throughput transcriptome analyses in this tissue [31]. This extremely high heterogeneity of transcript isoforms has been reported even in single RP genes (*CERKL* [32]). Of note, a very late report showed a neural-regulated alternative splicing program regulated by specific neuronal splicing factors, and associated to human neurological disorders [33]. In this context, it is worth mentioning that a pathogenic mutation in a retina-specific exon of *TTC8* (a gene otherwise causative of the Bardet-Biedl syndrome) exclusively causes RP [34].

Although network-based approaches are powerful tools to approach pathogenesis at the molecular basis, several challenges remain to be addressed. Some methodological issues deserve improvement, among them: the literature bias towards well-known proteins and genes; the high number of symbol aliases for the same gene/protein hampers the accuracy of the interaction retrieval; the filtering step over iHOP reference interactions has to be performed manually and thus, it is subjective; the computational restraints caused by the high number of interactions to consider; and finally, the development of new ways to visually integrate all the available information at the different levels to avoid clogging the graph generation.

On the other hand, the informativity and usefulness of networks increase with the addition of further relevant biological information. Most of the high-throughput data in repositories are based on cell line-based studies, which do not always reflect the subtle functional differences of highly specialized tissues, like specific neuronal tissues, such as the retina, or its highly specialized cells, such as photoreceptors. Besides, other relevant data should be also considered when building networks to understand etiopathogenesis, for instance: gene regulation at the epigenetics layer, transcriptional regulation by miRNAs and lnRNAs, different contribution of alternatively spliced isoforms, accurate subcellular protein localization and trafficking, all of which are currently expanding our view on gene/protein interactions and their relationship to the disease. Indeed, we contemplate to regularly refine our model by updating the core dataset with newly identified genes and novel interaction reports, to keep it a valuable resource for researchers working in RP and related retinal disorders.

## Conclusions

The great wealth of publicly available information either in the literature and high-throughput data repositories could be extremely useful to answer specific queries if big data approaches were properly customized to retrieve and process data. Although there are multiple generic online resources to build genetic and protein networks, our aim was: first, to generate a comprehensive and integrated dataset for a rare mendelian disorder and, second, develop a user-friendly web application for geneticists based on this curated dataset, RPGeNet. The differentiating traits of our approach are: i) the focus on the genes causing retinitis pigmentosa and their known interactors (the core network was built around the 110 RP/LCA genes reported so far); ii) the generation of a tailored subgraph (which allows a very specialized search, and a step-by-step visualization of the query interactors); iii) the submission of queries through any gene (RP and non-RP) located in the RP/LCA network (from the skeleton graph derived from the shortest paths between RP and LCA genes, to up to 2 levels of distance); and iv) the inclusion of relevant biological information at a glance through shape, color and size code of the nodes.

The exploratory data analysis through the RPGeNet interface facilitates the visualization of key molecular components linking RP genes. Bridging nodes are valuable to prioritize gene candidates within loci revealed by linkage analysis and identify putative pathogenic mutations out of the large amount of genetic variants revealed by exome sequencing. Overall, disease-based networks open new avenues to study etiopathogenesis, unveil valuable causative and disease-modifying candidates, and provide clues to approach therapy.

## Methods

### Gathering RP genes

The standard HUGO symbols [35] for the set of 110 RP/LCA genes were retrieved from RetNet [1], as well as the corresponding synonym identifiers. RetNet provides tables of genes and loci causing inherited retinal diseases. For this work, we have considered all non-syndromic retinitis pigmentosa (RP) and Leber congenital amaurosis (LCA) genes, plus selected syndromic genes with clear RP or LCA phenotypic traits according to RetNet. S1 Table summarizes information for those genes, which were used to define the core nodes of our interactions network. This table contains links to their corresponding genomic location (human genome hg19 at UCSC genome browser [36]), the Online Mendelian Inheritance in Man (OMIM [37]), NCBI Gene [38] and RefSeq [39], as well as UniProt [40] databases. The RP/LCA causative genes follow different Mendelian inheritance patterns (dominant, autosomal recessive, and X-linked), and are involved in multiple cellular processes, as described in Table 1, where other genetic details are also provided (e.g. genes involved in both syndromic and non-syndromic RP/LCA; genes causing several retinal dystrophies).

**Table 1:** A summary of the functional annotation for RP/LCA driver genes.

Functional Annotation	Syndromic RP/LCA	Non-syndromic RP/LCA
Development of retina and or its components	*CLRN1* *, *GDF6* *, *NEUROD1* *, *OTX2*	*C2ORF71*, *ZNF408* ^#^, *ZNF513*
Photoreceptor specific transcription factor		*CRX* ^#^, *NRL*, *NR2E3* ^#^
Phototransduction		*CNGA1*, *CNGB1*, *GUCY2D*, *PDE6A*, *PDE6B* ^#^, *PDE6G*, *PRPH2* ^#^, *RHO* ^#^, *SAG* ^#^,
Vitamin A (retinol) metabolism	*RDH11*	*ABCA4* ^#^, *LRAT* ^#^, *RGR* ^#^, *RDH12* ^#^, *RLBP1* ^#^, *RPE65* ^#^
Lipid synthesis/modification	*INPP5E*	*CYP4V2* ^#^, *DHDDS*, *MVK*
Structural or cytoskeletal	*BBS1* *, *BBS2* *, *OFD1* *	*EMC1*, *EYS*, *FSCN2* ^#^, *IMPG2*, *PROM1* ^#^, *ROM1*, *RP1*, *TULP1* ^#^
Signaling, cell-cell interactions, or synaptic interaction	*ABHD12*, *TUB*, *USH2A* *	*CABP4*, *CRB1* ^#^, *GUCA1B* ^#^, *RP2*, *SEMA4A* ^#^
RNA intron-splicing process		*DHX38*, *PRPF3*, *PRPF4*, *PRPF6*, *PRPF8*, *PRPF31*, *RP9*, *SNRP200*
Formation/maintenance of ciliated cells	*AHI1*, *ARL6* *, *CC2D2A*, *CEP290* *, *IQCB1* *, *INVS*, *NPHP1*, *NPHP3*, *NPHP4*, *SDCCAG8*, *TTC8* *	*FAM161A*, *LCA5*, *RPGR* ^#^, *RP1L1* ^#^
Spindle formation	*IFT172* *	*KIZ*, *NEK2*
Ubiquitin/SUMO pathways		*KLHL7*, *TOPORS*
pH regulation		*CA4*
Transport	*FLVCR1*	*AIPL1* ^#^, *ARL2BP*, *RD3* ^#^, *RBP3*, *SLC7A14*
Phosphorylation	*PANK2*, *PRPS1*	*HK1*, *MAK*
Peroxisomal biogenesis/import	*HSGNAT* *, *PEX1*, *PEX2*, *PEX7*	
Phagocytosis		*MERTK* ^#^
Orphan receptor		*GPR125*
Olfactory receptor		*OR2W3*
Other/unknown functions	*DTHD1* *, *GNPTG*, *PHYH*, *TTPA*	*BEST1* ^#^, *C8ORF37* ^#^, *CERKL* ^#^, *IDH3B*, *IMPDH1* ^#^, *KCNJI3* ^#^, *KIAA1549*, *NMNAT1*, *PRCD* ^#^, *RPGRIP1* ^#^, *SPATA7* ^#^

* Mutations in this gene can cause both syndromic and non-syndromic forms of RP or LCA

^#^ Mutations in this gene can cause RP as well as other retinal dystrophies

### Collecting interactions data

Once all the genes were retrieved from RetNet, a search was performed to identify genes or proteins that interacted with our RP/LCA genes in a directed manner and also find those genes/proteins the driver genes interact with (considering that the interactions are directional and binary). Therefore, different suitable datasets were selected. A hand-curated list of interacting partners was retrieved by searching for a subset of 62 selected non-syndromic RP gene symbols over the iHOP interactions database [23,41], which is based on relationships mined from the scientific literature. A table of 141 confident interaction partners was manually built after carefully inspecting the sentences provided by the iHOP search engine; this table defined 117 edges that link 95 nodes.

On the other hand, in an attempt to recover the most updated gene and protein relationships information, we syntactically parsed interaction sentences filtered out from PubMed abstracts as well as the full text electronic versions (ePub) when available (Castillo-Lara and Abril, manuscript in preparation). The initial query “*retinitis pigmentosa*” (on October 1^st^, 2014), restricted to “*Journal articles*” on PubMed server, returned 8,768 abstract hits, of which 1,204 had full-text available; from those 8,768 hits, we were able to retrieve the abstract only for 6,251. This approach is referred throughout the manuscript as “Sparser” because it is based on the Stanford parser (version 3.5.0 [42]). This tool was used to analyze 54,185 and 347,034 sentences, for abstract and full-text, respectively, resulting on 64 and 628 raw interactions (60 and 440 distinct interactions). The first two Venn diagrams on S2 Fig show the overlap across nodes and edges identified by this approach.

High-throughput interaction data were retrieved from BioGRID (version 3.2.117 [20,21]) and STRING (Search Tool for the Retrieval of Interacting Genes/Proteins, version 9.1 [22]). Raw pair-wise protein and gene interactions were downloaded from the former: 15,394 nodes connected by 159,034 edges were retrieved out of 232,888 human-specific interactions (this database stores 761,693 total interactions). On the other hand, STRING focuses on known and predicted protein-protein interaction (PPIs): 15,999 nodes and 640,679 edges were considered out of 926,130 human-specific interactions (database has 114,926,847 total interactions).

### Reconstructing the RP interactions network

A Perl script was implemented to capture the interactions subnetwork based on a set of reference genes, in our case the RP/LCA genes. In order to disambiguate node names and ensure the maximum number of occurrences for the driver genes, once a whole interaction set was collected, all the symbols were mapped into the standard name by using tables of aliases retrieved also from the interactions databases and verified against HGNC symbol tables. RP/LCA genes were then used to find all the possible pair-wise shortest paths by applying the Dijkstra algorithm implemented in the Graph Perl module. Graph::Directed module was used to define the whole network data structure as a directed graph, which simplified the calculations for the RP/LCA subnetwork.

The script produced a skeleton graph and up to four expansion levels; each expanded level included those nodes at distance one of the current level nodes (namely new parent and child nodes). Some of the RP/LCA genes connections (considered here as the node degree) saturated earlier than others, as it can be observed on S3 Table. Graphs were stored in three different standard formats: GraphViz simplified dot format to facilitate automated analyses—GraphViz commands were used in early steps to visualize the nets—; graphml to store information about edges, such as supporting evidences and database of origin; and finally, JSON to make data available on the RPGeNet web interface [19].

Dot files were processed using Gephi [43] to compute statistics on the graphs and visualize whole-networks at distinct levels. For drawing those networks, Yifan Hu proportional [44] followed by Force Atlas 2 [45] layout algorithms were applied, refined by label adjust and/or by weighting nodes according to their degree. The web interface was developed for user-friendly network exploration to RP researchers. It was implemented via PHP scripts to process queries, integrate the data and display the resulting network through the open-source Cytoscape JavaScript library for graph analysis and visualization [46]. Further graph calculations to analyze the contribution of each distinct dataset to the main RP network were performed using the igraph R package [47].

The main web form provides two entry points, one focuses on selected genes (similarly to other current gene/protein browsers), and the second recovers the paths containing a given RP/LCA gene within the RPGeNet. The network display (see Figs 4–6) facilitates the interaction with the nodes by zooming, displacing, changing the graph layout—grid, random, circle, breadthfirst, cose, concentric, and so on—, adding or removing nodes, and retrieving information about RP/LCA genes. Instead of adjusting the node sizes to their degree as a measure of connectivity, we chose to depict the total number of variants (polymorphisms) reported for each locus [48]. The border color encodes information about the degree of distance to the closest RP/LCA gene. Driver genes are always colored in violet when connected, or in orange if not. “Parent” nodes distance—up to four levels—is encoded by green shades whereas “child” nodes take blue shades, the only exception being the skeleton graph, where all the non-RP nodes use grey border colors. The shape of the nodes is a circle by default, but square nodes pinpoint syndromic RP/LCA genes. Besides, the filling core of the nodes encode tissue specific gene expression, which was retrieved from the Gene Expression Omnibus (GEO [49]). Series GSE7905 contains microarray data for 31 human tissues (three replicates each), which were used to create the Applied Biosystems Human BodyMap (Tissue Gene Expression Database). For this specific task we run a standard protocol based on Bioconductor [50] limma R package. After normalization of the expression levels, retina expression levels were compared against the average tissue expression in order to find over- or under-expressed genes in retinal tissue under normal conditions. In the future, this feature could also reflect expression data from distinct sources.

### Availability of supporting data

Apart from the supplementary files, further material is provided at the RPGeNet web page [19]. The developed software is available upon request.

## Supporting Information

S1 Fig Syntactical analysis performed to capture interactions from RP/LCA-specific literature. Upper panel shows the distribution of the number of interactions retrieved from journal articles, processed either at the abstract or full-text levels, in orange and purple respectively. The electronic full-text could be retrieved only for 1/8^th^ of the documents that had an abstract. Of interest, there is a clear increase in the interactions reported in the recent years. The bottom panel depicts the complexity of the sentences describing a gene/protein interaction. The number of sentences is summarized on each circle; the level in the sentence parsing-tree was recorded for the subject and predicate containing a gene/protein symbol identifier. The x-axis represents the substraction value among the subject and predicate levels, while the y-axis value is the average of the absolute value of those two levels. Complex sentences push spots up; therefore, a cut-off of average level above 4.5 can be set to discard false positive interactions. It is worth to note that 75% of the sentences were comprised within this range.
(PDF)

S2 Fig Reconstructing a preliminary RP interaction network from different sources. A graph skeleton was produced for every interactions dataset separately, using a set of 62 non-syndromic RP genes as bait to capture the paths connecting all of them, in order to assess the individual contribution of each dataset and to illustrate the complementarity of the approaches for the different datasets. The overlap graph is shown for five different dataset combinations (from left to right and from top to bottom): 1) interactions filtered by Sparser from abstracts, having or not full text available (Abs and EpubAbs respectively); 2) Sparser interactions for all abstracts (FullAbs) compared with those retrieved from full text (Epub); 3) overlap among interactions filtered from iHOP and manually curated, from those provided by versus Sparser interactions; 4) skeleton comparison for the two high-throughput (HT) databases, BioGRID and STRING; and finally, 5) the triple merge of skeleton graphs for iHOP curated interactions, Sparser extraction, and HT databases. Different node colors are correlated to the sets depicted on the Venn diagrams, close to each graph plot. Square nodes refer to the 62 RP genes used as bait to distill the subnetworks. RP_C_ value corresponds to the connected RP genes, those that were found on the network when distilling the skeleton subnetwork. It can be observed that this number increases when considering larger interactions sets, but also varies when merging the individual subnetworks. The largest RP_C_ value (56) is retrieved when combining all the interactions from the different datasets in a single run, which was used to distill the skeleton graph underlying the RPGeNet browser.
(PDF)

S3 Fig Pairwise connectivity of RP/LCA genes. Each of the diagrams show the pair-wise connectivity between RP/LCA genes or proteins from the interaction network generated, where *d* corresponds to the distance for the shortest directed path across the network between the pair of connected driver genes—maximum distance found was of 6 edges (or five internal nodes between the two RP/LCA genes)—. The last plot summarizes all the previous diagrams, showing all the possible connections among pairs of query genes at different distances. On the outer ring we have depicted the standard symbols for the genes used to build the network (S3-A: 110 genes, both non-syndromic and syndromic; S3-B: 75 non-syndromic genes; S3-C: 35 syndromic genes), they were sorted first by the number of incoming, then by the outcoming connections, and finally following an alphabetical order. Each of the driver genes that interact with other RP/LCA genes has a distinct color box, while those genes for which there was no reported interaction are shown in black. The colored boxes are divided in two moieties: the lighter half groups all the outgoing connections—thus reflecting a directed path starting from the current RP node—, whereas the darker half gathers all the incoming connections—indicating a directed path ending at the current RP node. See further details on Fig 3. All diagrams on this figure were made using Circos [28].
(PDF)

S1 Table Summary of the 110 genes related to RP/LCA phenotypic inheritance. The genes were selected from RetNet [1] and classified into four Mendelian inheritance categories, of which the first contains six genes that depending on the mutation studied present a dominant or a recessive phenotype. Reference HUGO symbol notation was used as gene identifier across all the analyses. Location column shows the starting coordinate on a chromosome and the gene strand for the longest transcript isoform of the gene annotated over the h19 human genome version. Those genomic locations are linked to the UCSC genome browser [36]. The other columns show the identifiers linked to the corresponding entry on the OMIM [37], NCBI-Gene [38], NCBI-RefSeq [39], and UniProt [40] databases.
(PDF)

S2 Table Basic graph statistics for the RP/LCA genes interaction networks. Comparison of the number of nodes (gene/protein) and edges (interactions) provided by the different datasets considered, as well as four intersections: joining interactions filtered from literature abstracts and full text (“Sparser ALL”); its combination with iHOP curated interactions (“Sparser + iHOP”); the merge of high-throughput data (“BioGRID + STRING”); and the result of processing all together (“ALL DBs”). “*Hsap* Nodes” were filtered from BioGRID human-specific interactions. “Unique Nodes/Edges” correspond to the simplified set of interactions that define the integrated graph from which the RP/LCA network was filtered out. “Unique Nodes” also include the RP/LCA genes that had no reported interactions on each dataset. Last three columns summarize the statistics for the networks computed over three sets of driver genes: non-syndromic, syndromic, or both (all). The latter constitutes the core of the RPGeNet webapp. Graph statistics are described in depth on [51].
(PDF)

S3 Table Barplot of the connectivity for the RP/LCA genes showing the network saturation. Names of syndromic RP/LCA genes are marked in red, to distinguish from the non-syndromic ones. Conditional formatting on the cells in the spread sheet produce three horizontal barplots depicting the RP genes connectivity (“INDEGREE” for the incoming edges, “OUTDEGREE” for the outcoming, and “DEGREE” for the sum), based only on distinct interactions. Each barplot scale corresponds to five columns of the spread sheet, from levels zero (the skeleton graph) to 4 (the maximum graph level computed, yet webapp can only handle up to the level 2). Cells are filled in blue when the value on the left is smaller than the current cell value (therefore, the connectivity increases with the level). Note that for all RP/LCA genes connectivity saturates at level one; due to the methodology applied to build the skeleton network all their interactors are already included in that level. On the other hand, as expected, the total number of graph nodes and edges increases with the graph level (see bottom rows). RP_F_ sums up the RP genes having at least one connection at each column. “Max” and “Avg” degree indicate the maximum and average degree, respectively; it is worth mentioning that the average indegree was 9.87 and the outdegree was 12.22 for the skeleton network. The red marks identify those RP genes that reach a connectivity degree greater than the maximum value of their average degree (59.58). Several genes are highlighted for both high indegree and outdegree connectivities; among those, one can mention: a centrosomal protein 290kDa (*CEP290*), a cone-rod homeobox protein (*CRX*), a DEAD-box protein probably involved in splicing (*DHX38*), the isocitrate dehydrogenase 3 beta subunit (*IDH3B*), several kinases (*HK1*, *MAK*, *MERTK*, and), several splicing splicing factors (*PRPF3*, *PRPF4*, *PRPF6*, *PRPF8*, *PRPF31*, and *SNRNP200*), rhodopsin (*RHO*), and an E3 ubiquitin-protein ligase (*TOPORS*).
(PDF)

S4 Table Summary of the RP/LCA genes interactions identified from different sources. Number of interactions found when at least one of the partners is an RP/LCA gene. Names of syndromic RP/LCA genes are marked in red, to distinguish from the non-syndromic ones. Rows at the bottom (6) sum up the number of nodes and interactions. Number of nodes are indicated as follows: RP_F_ for RP genes found, and RP_M_ for those missing in the data set; RP_C_skel_ for those connected to another RP gene via the shortest path used to build the skeleton graph (level 0); and finally, RP_C_level1_ for RP genes integrated into the graph level 1 (as “parents” or “children” of a non-RP gene from the skeleton graph). “Total RP interactions” summarizes all the interactions found on each data source, and “Total DB interactions” indicates the global number of interactions contained in those sources. In all columns, “Sparser” corresponds to the Stanford-parser syntactical analysis approach. “Sparser ALL” shows the number of interactions found when merging (but not directly adding) the two input datasets defined for “Sparser Abstracts” and “Sparser FullText”. The same applies to the combined approaches: “Sparser + iHOP”, “BIOGRID + STRING”, and “ALL DBs”. The latter contains the resulting interactions found for each RP gene when all the five sets were used as input of the graph distiller program. While the previous columns show results based on a preliminary analysis of 62 RP/LCA genes, last three columns—shaded in light green—show the numbers for the gene sets used as driver genes for the RPGeNet, split into non-syndromic RP/LCA genes, RP/LCA syndromic genes, and both lists used together.
(PDF)
