# Supplementary figures and images for: Distilling a Visual Network of Retinitis Pigmentosa Gene-Protein Interactions to Uncover New Disease Candidates
Source: PLoS One. 2015 Aug 12;10(8):e0135307. doi: 10.1371/journal.pone.0135307 (PMC4534355; doi:10.1371/journal.pone.0135307)

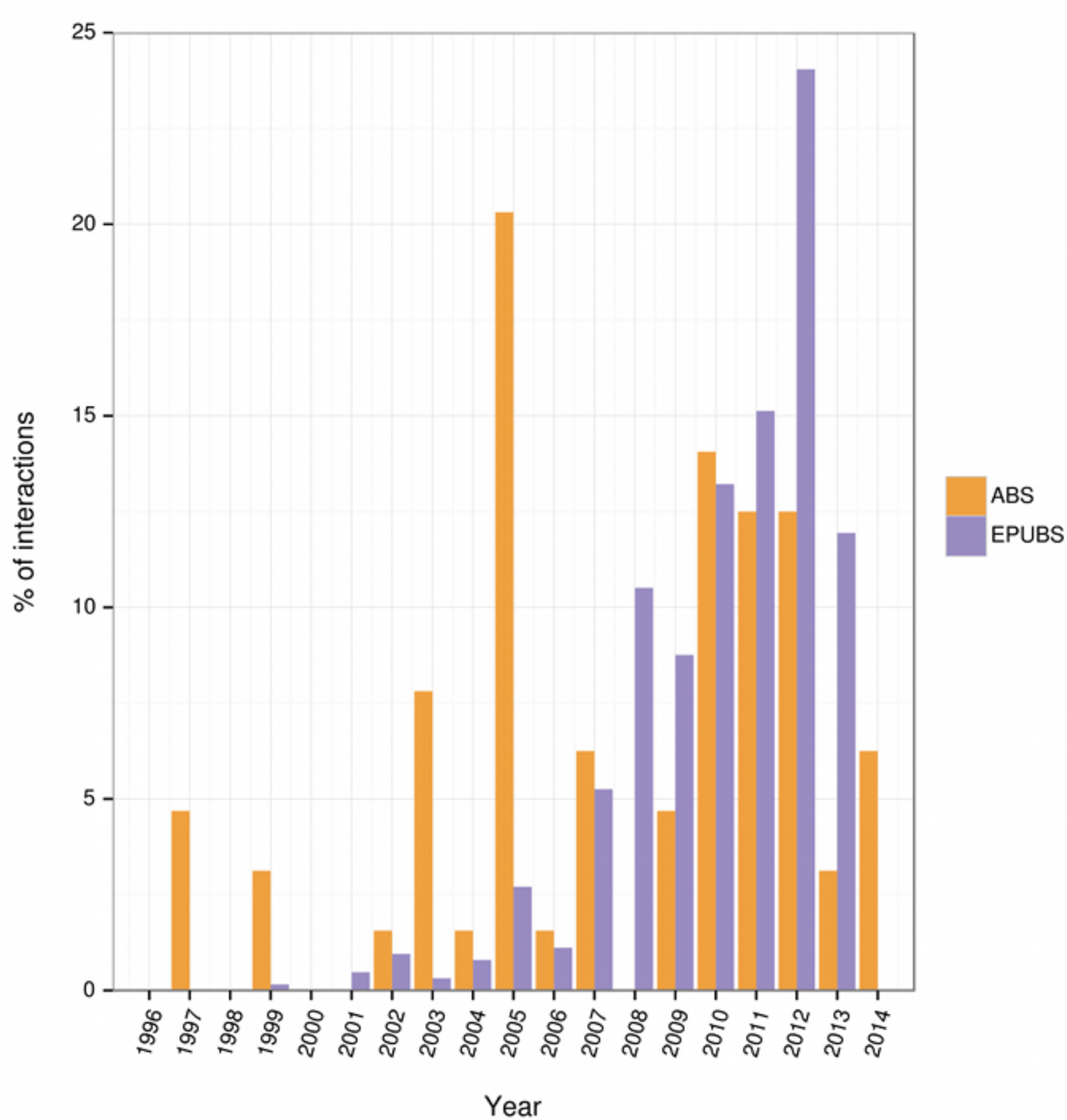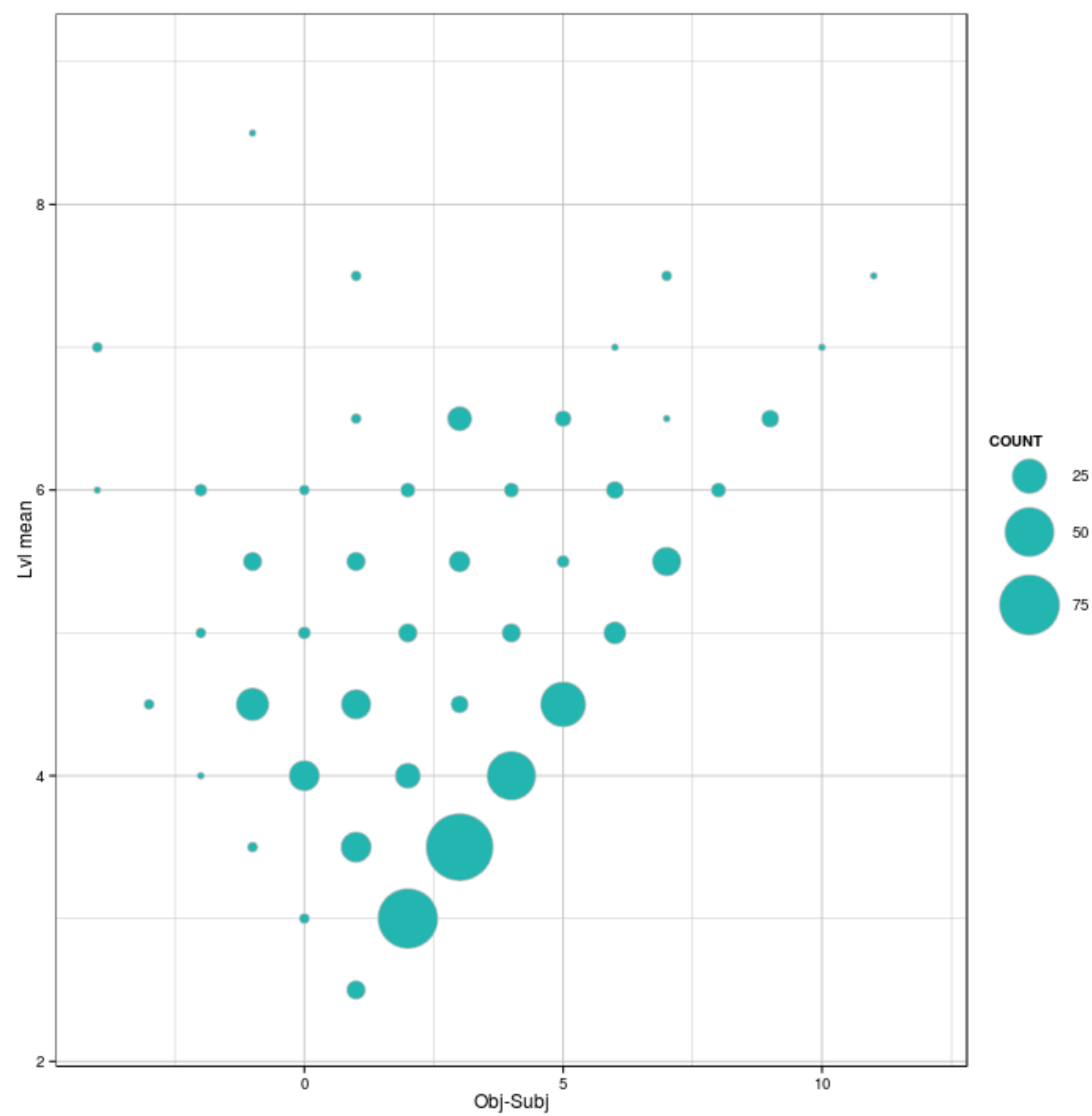

Supplement: S1 Fig — Upper panel shows the distribution of the number of interactions retrieved from journal articles, processed either at the abstract or full-text levels, in orange and purple respectively. The electronic full-text could be retrieved only for 1/8th of the documents that had an abstract. Of interest, there is a clear increase in the interactions reported in the recent years. The bottom panel depicts the complexity of the sentences describing a gene/protein interaction. The number of sentences is summarized on each circle; the level in the sentence parsing-tree was recorded for the subject and predicate containing a gene/protein symbol identifier. The x-axis represents the substraction value among the subject and predicate levels, while the y-axis value is the average of the absolute value of those two levels. Complex sentences push spots up; therefore, a cut-off of average level above 4.5 can be set to discard false positive interactions. It is worth to note that 75% of the sentences were comprised within this range. (PDF) [file pone.0135307.s001.pdf]
