# Supplementary material for: Distilling a Visual Network of Retinitis Pigmentosa Gene-Protein Interactions to Uncover New Disease Candidates
Source: PLoS One. 2015 Aug 12;10(8):e0135307. doi: 10.1371/journal.pone.0135307 (PMC4534355; doi:10.1371/journal.pone.0135307)
Supplement: S2 Fig — A graph skeleton was produced for every interactions dataset separately, using a set of 62 non-syndromic RP genes as bait to capture the paths connecting all of them, in order to assess the individual contribution of each dataset and to illustrate the complementarity of the approaches for the different datasets. The overlap graph is shown for five different dataset combinations (from left to right and from top to bottom): 1) interactions filtered by Sparser from abstracts, having or not full text available (Abs and EpubAbs respectively); 2) Sparser interactions for all abstracts (FullAbs) compared with those retrieved from full text (Epub); 3) overlap among interactions filtered from iHOP and manually curated, from those provided by versus Sparser interactions; 4) skeleton comparison for the two high-throughput (HT) databases, BioGRID and STRING; and finally, 5) the triple merge of skeleton graphs for iHOP curated interactions, Sparser extraction, and HT databases. Different node colors are correlated to the sets depicted on the Venn diagrams, close to each graph plot. Square nodes refer to the 62 RP genes used as bait to distill the subnetworks. RPC value corresponds to the connected RP genes, those that were found on the network when distilling the skeleton subnetwork. It can be observed that this number increases when considering larger interactions sets, but also varies when merging the individual subnetworks. The largest RPC value (56) is retrieved when combining all the interactions from the different datasets in a single run, which was used to distill the skeleton graph underlying the RPGeNet browser. (PDF) [file pone.0135307.s002.pdf]

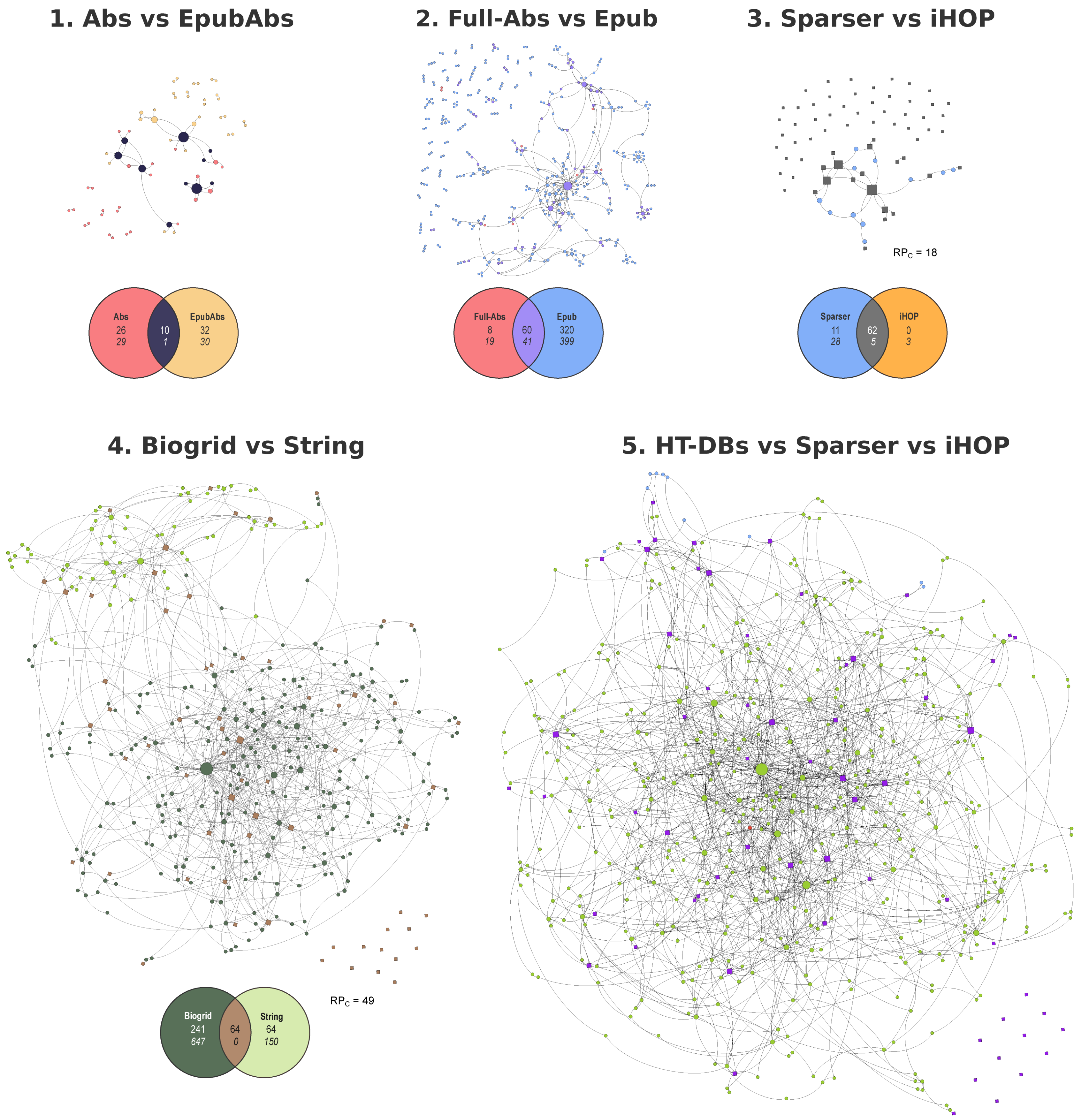

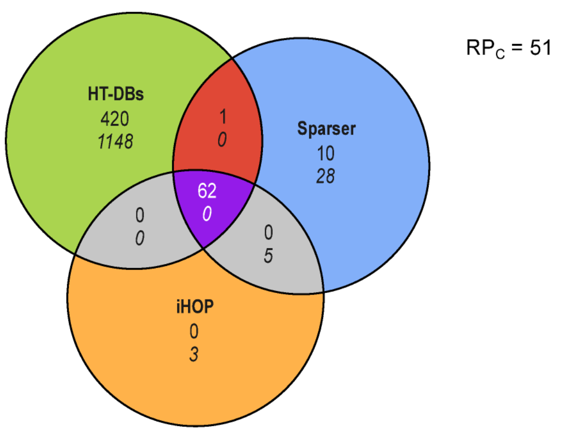

Reconstructing the RP interactions network from different sources.

S2 Fig. Boloc et al, *PLoS ONE*, 2015.

A graph skeleton was produced for every interactions dataset separately, using the RP genes as bait to capture the paths connecting all of them, in order to assess the individual contribution of each dataset and to illustrate the complementarity of the approaches for the different datasets. The overlap graph is shown for five different dataset combinations (from left to right and from top to bottom): 1) interactions filtered by Stanford parser (Sparser) from abstracts, having or not full text available (`Abs` and `EpubAbs` respectively); 2) Sparser interactions for all abstracts (`FullAbs`) compared with those retrieved from full text (`Epub`); 3) overlap between interactions filtered from iHOP and manually curated, from those provided by versus Sparser interactions; 4) skeleton comparison for the two high-throughput (HT) databases, BioGRID and STRING; and finally, 5) the triple merge of skeleton graphs for iHOP curated interactions, Sparser extraction, and HT databases. Different node colors are correlated to the sets depicted on the Venn diagrams, close to each graph plot. The analysis shown in this figure was run on a preliminary set of 62 RP genes used as bait to distill the subnetworks, those genes are represented as square nodes on the graphs. RPC value corresponds to the number of connected RP genes, those that were found on the network when distilling each skeleton subnetwork. It can be observed that this number increases when considering larger interactions sets, but also varies when merging the individual subnetworks. The largest RPC value (56) is retrieved when combining all the interactions from the different datasets in a single run, which was used to distill the skeleton graph underlying the RPGeNet browser. Figure produced using Inkscape [`http://inkscape.org/`] over a set of graphs generated by Gephi [`http://gephi.github.io/`].
