# Supplementary material for: Distilling a Visual Network of Retinitis Pigmentosa Gene-Protein Interactions to Uncover New Disease Candidates
Source: PLoS One. 2015 Aug 12;10(8):e0135307. doi: 10.1371/journal.pone.0135307 (PMC4534355; doi:10.1371/journal.pone.0135307)
Supplement: S3 Fig — Each of the diagrams show the pair-wise connectivity between RP/LCA genes or proteins from the interaction network generated, where d corresponds to the distance for the shortest directed path across the network between the pair of connected driver genes—maximum distance found was of 6 edges (or five internal nodes between the two RP/LCA genes)—. The last plot summarizes all the previous diagrams, showing all the possible connections among pairs of query genes at different distances. On the outer ring we have depicted the standard symbols for the genes used to build the network (S3-A: 110 genes, both non-syndromic and syndromic; S3-B: 75 non-syndromic genes; S3-C: 35 syndromic genes), they were sorted first by the number of incoming, then by the outcoming connections, and finally following an alphabetical order. Each of the driver genes that interact with other RP/LCA genes has a distinct color box, while those genes for which there was no reported interaction are shown in black. The colored boxes are divided in two moieties: the lighter half groups all the outgoing connections—thus reflecting a directed path starting from the current RP node—, whereas the darker half gathers all the incoming connections—indicating a directed path ending at the current RP node. See further details on Fig 3. All diagrams on this figure were made using Circos [28]. (PDF) [file pone.0135307.s003.pdf]

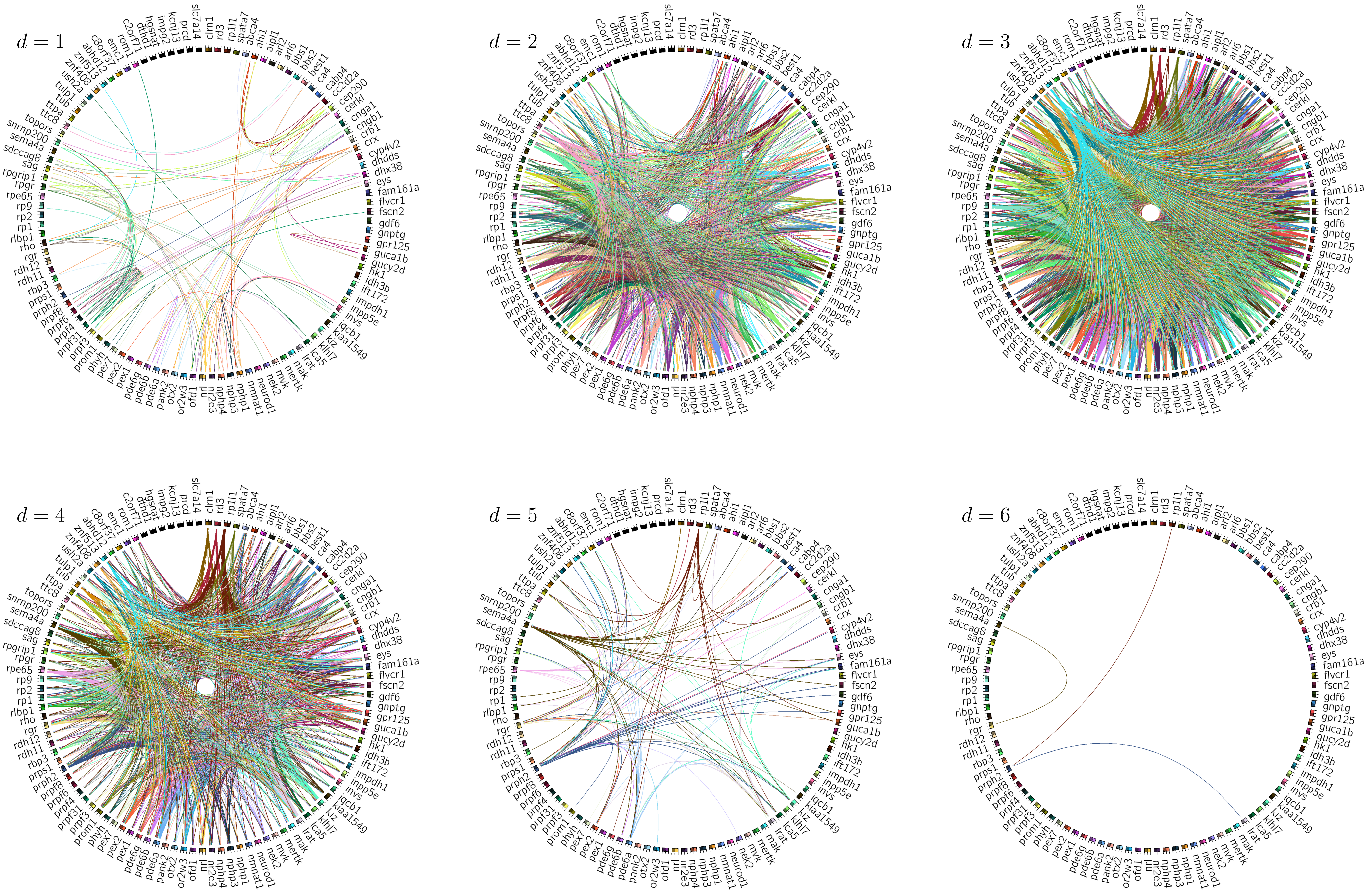

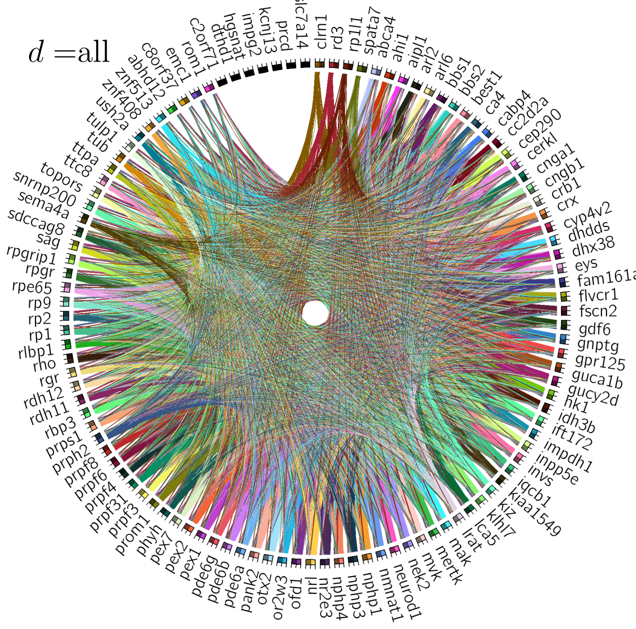

Supplementary Figure S3 (A) - Boloc et al, *PLoS One, 2015.*
RP genes pair-wise connectivity (all)
Each of the diagrams show the pair-wise connectivity between RP genes or proteins from the interaction network generated, where d corresponds to the distance for the shortest directed path across the network between the pair of connected RP genes—maximum distance found was of 6 edges (or five internal nodes between the two RP genes)—. The last plot summarizes all the previous diagrams, showing all the possible connections among pairs of RP genes at different distances. On the outer ring we have depicted the standard symbols for the 110 RP/ genes used to build the network, they were sorted by the number of incoming connections, then by the outcoming ones. Each of the RP genes found to interact with other RP genes has a distinct color box, while those genes for which there was not interaction found are shown in black. The color boxes have two halves: one lighter grouping all the outgoing connections—so there is a directed path from current gene node—, and one darker gathering all the incoming connections—meaning that there is a directed path to current gene node—. By making this difference it is easy to spot genes that are upstream of many in the network—like *CLRN1*, *RD3*, *RP1L1*, and *SPATA7*—, mainly downstream of others—like *ABHD12*, *ROM1*, *EMC1*, and *C8orf37*—, or even internal hubs—like *PEX1*, *IQCB1*, *CERKL*, *CRX* and *RP2*—. All diagrams made using Circos[1].

[1]Krzywinski *et al.* "Circos: An information aesthetic for comparative genomics." *Genome Research*, 19(9):1639–1645, 2009.

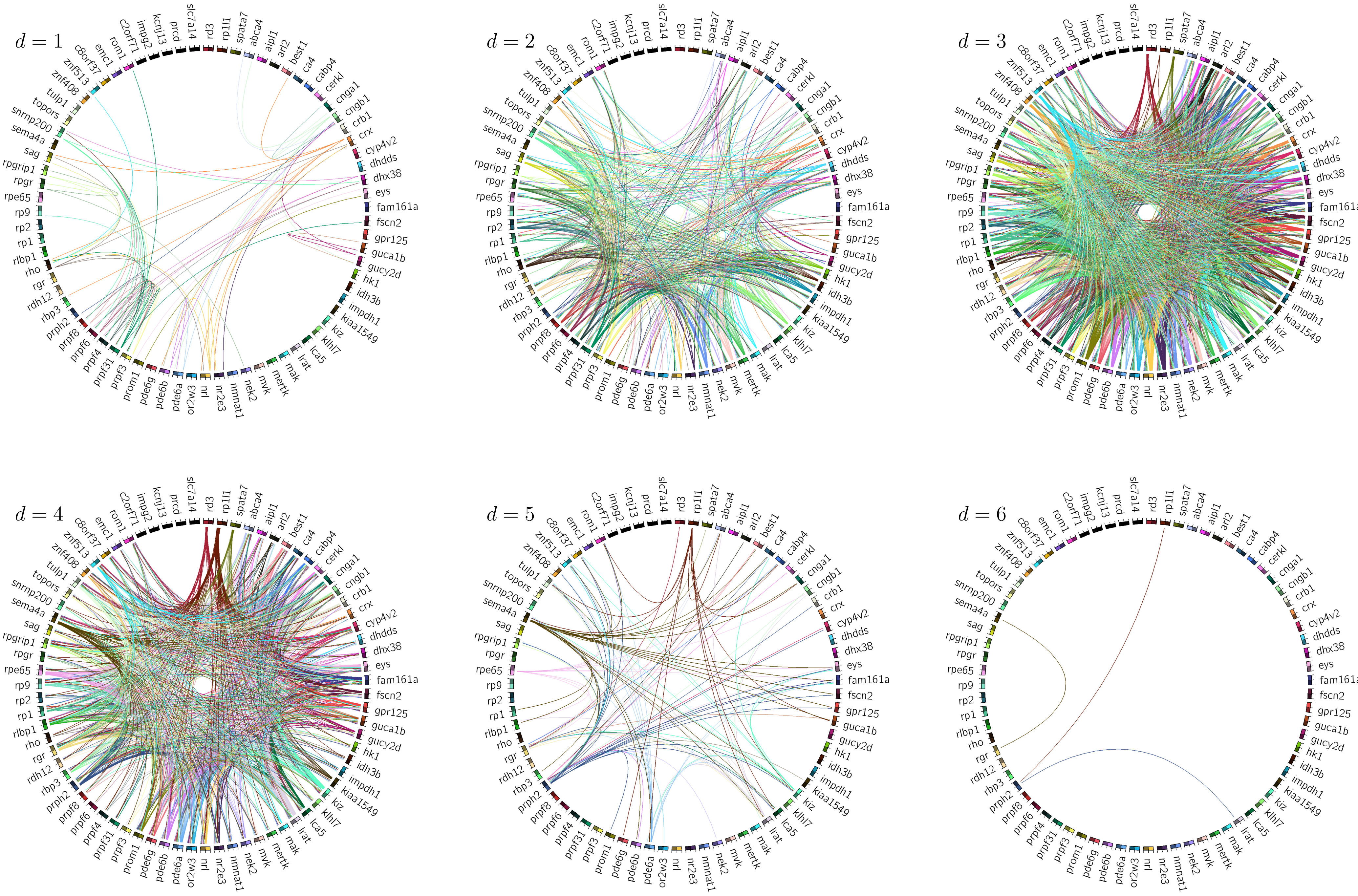

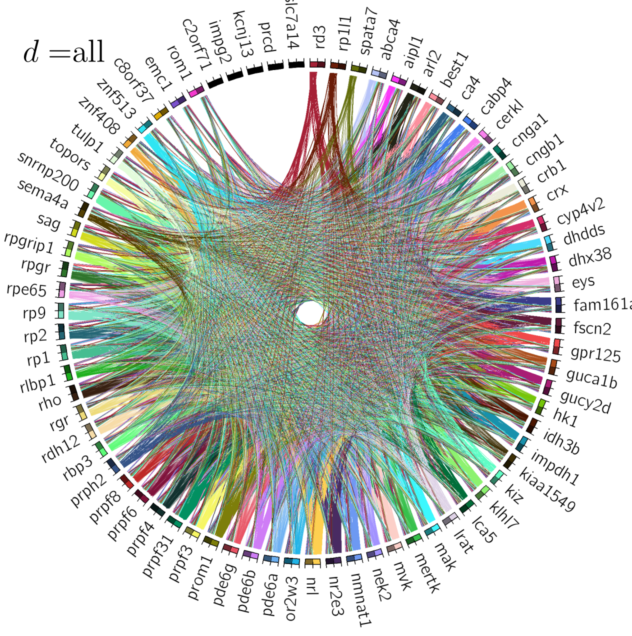

Supplementary Figure S3 (B) - Boloc et al, *PLoS One, 2015.*
RP genes pair-wise connectivity (non-syndromic)
Each of the diagrams show the pair-wise connectivity between RP genes or proteins from the interaction network generated, where d corresponds to the distance for the shortest directed path across the network between the pair of connected RP genes—maximum distance found was of 6 edges (or five internal nodes between the two RP genes)—. The last plot summarizes all the previous diagrams, showing all the possible connections among pairs of RP genes at different distances. On the outer ring we have depicted the standard symbols for the 75 non-syndromic RP genes used to build the network, they were sorted by the number of incoming connections, then by the outcoming ones. Each of the RP genes found to interact with other RP genes has a distinct color box, while those genes for which there was not interaction found are shown in black. The color boxes have two halves: one lighter grouping all the outgoing connections—so there is a directed path from current gene node—, and one darker gathering all the incoming connections—meaning that there is a directed path to current gene node—. By making this difference it is easy to spot genes that are upstream of many in the network—like *RD3*, *RP1L1*, and *SPATA7*—, mainly downstream of others—like *ROM1*, *EMC1*, and *C8orf37*—, or even internal hubs—like *CERKL*, *CRX* and *RP2*—. All diagrams made using Circos[1].

[1] Krzywinski *et al.* "Circos: An information aesthetic for comparative genomics." *Genome Research*, 19(9):1639–1645, 2009.

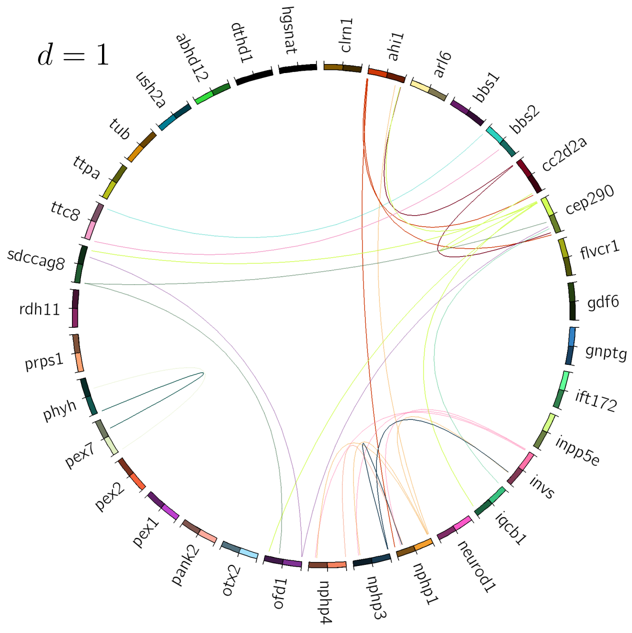

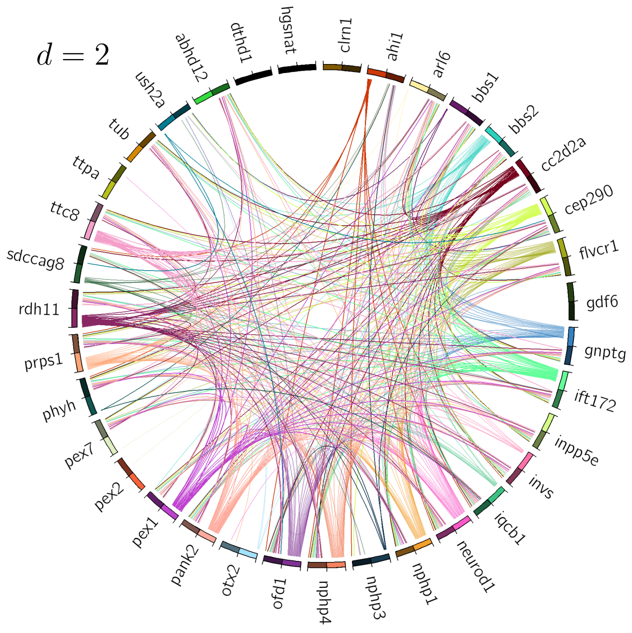

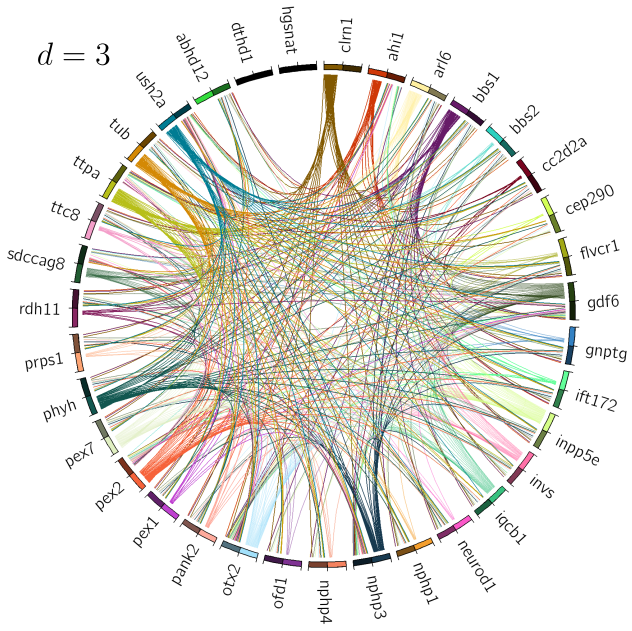

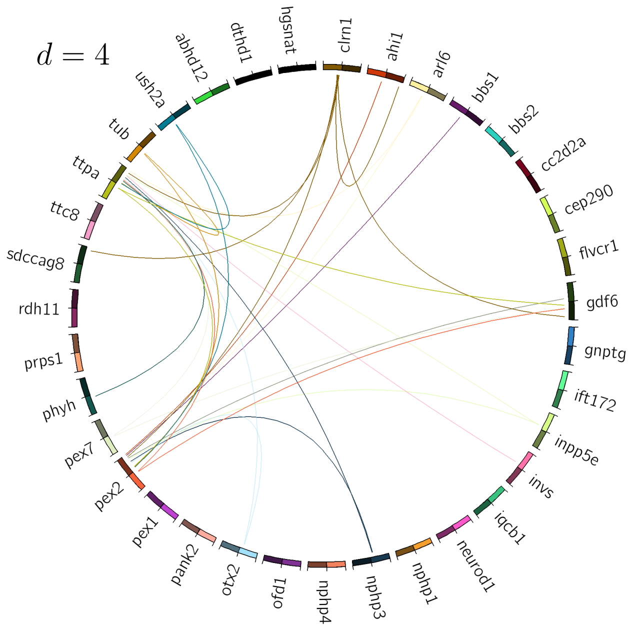

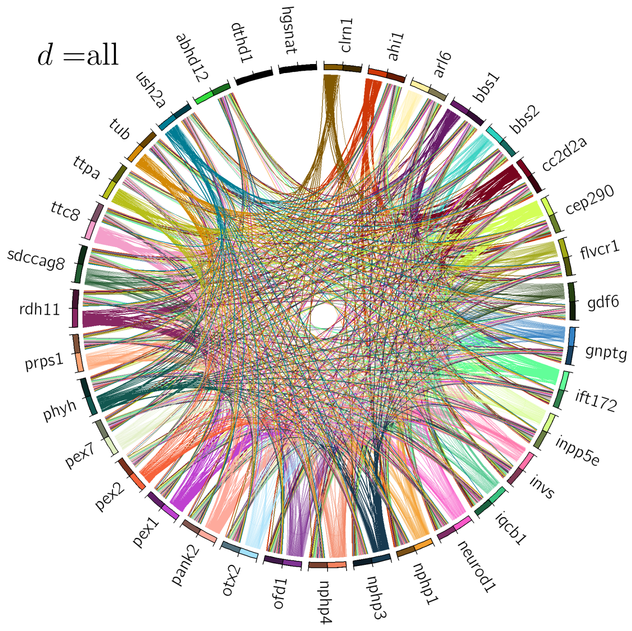

Supplementary Figure S3 (C) - Boloc et al, *PLoS One, 2015.*

RP genes pair-wise connectivity (syndromic)

Each of the diagrams show the pair-wise connectivity between RP genes or proteins from the interaction network generated, where d corresponds to the distance for the shortest directed path across the network between the pair of connected RP genes—maximum distance found was of 4 edges (or three internal nodes between the two RP genes)—. The last plot summarizes all the previous diagrams, showing all the possible connections among pairs of RP genes at different distances. On the outer ring we have depicted the standard symbols for the 35 syndromic RP genes used to build the network, they were sorted by the number of incoming connections, then by the outcoming ones. Each of the RP genes found to interact with other RP genes has a distinct color box, while those genes for which there was not interaction found are shown in black. The color boxes have two halves: one lighter grouping all the outgoing connections—so there is a directed path from current gene node—, and one darker gathering all the incoming connections—meaning that there is a directed path to current gene node—. By making this difference it is easy to spot genes that are upstream of many in the network—like *CLRN1*—, mainly downstream of others—like *ABHD12*—, or even internal hubs—like *PEX1* or *IQCB1*—. All diagrams made using Circos[1].

[1]Krzywinski *et al.* "Circos: An information aesthetic for comparative genomics." *Genome Research*, 19(9):1639–1645, 2009.
