# Supplementary material for: Distilling a Visual Network of Retinitis Pigmentosa Gene-Protein Interactions to Uncover New Disease Candidates
Source: PLoS One. 2015 Aug 12;10(8):e0135307. doi: 10.1371/journal.pone.0135307 (PMC4534355; doi:10.1371/journal.pone.0135307)
Supplement: S1 Table — The genes were selected from RetNet [1] and classified into four Mendelian inheritance categories, of which the first contains six genes that depending on the mutation studied present a dominant or a recessive phenotype. Reference HUGO symbol notation was used as gene identifier across all the analyses. Location column shows the starting coordinate on a chromosome and the gene strand for the longest transcript isoform of the gene annotated over the h19 human genome version. Those genomic locations are linked to the UCSC genome browser [36]. The other columns show the identifiers linked to the corresponding entry on the OMIM [37], NCBI-Gene [38], NCBI-RefSeq [39], and UniProt [40] databases. (PDF) [file pone.0135307.s004.pdf]

S1 Table. Summary of the 110 genes related to RP/LCA phenotype inheritance. The genes were selected from RetNet [1] and classified into four Mendelian inheritance categories, of which the first contains six genes that depending on the mutation studied present a dominant or a recessive phenotype. Reference HGNC[2,3] symbol notation was used as gene identifier across all the analyses. Gene symbols in red correspond to 35 selected syndromic RP/LCA genes, the rest of symbols are in black and comprise 75 non-syndromic RP/LCA genes. Location column shows the starting coordinate on a chromosome and the gene strand for the longest transcript isoform of the gene annotated over the h19 human genome version. Those genomic locations are linked (one can click on them) to the UCSC genome browser [4]. The other columns show the identifiers linked to the corresponding entry on the OMIM [5], NCBI-Gene [6], NCBI-RefSeq [7], and UniProt [8] databases.

1. RetNet, the Retinal Information Network. . http://sph.uth.edu/retnet/
2. HUGO Gene Nomenclature Commitee. http://www.genenames.org/
3. Gray KA, Yates B, Seal RL, Wright MW, Bruford EA. Genenames.org: the HGNC resources in 2015. Nucleic Acids Res. 2015; 43(Database issue):D1079-85. doi:10.1093/nar/gku1071
4. Karolchik D, Barber GP, Casper J, Clawson H, Cline MS, Diekhans M, et al. The UCSC Genome Browser database: 2014 update. Nucleic Acids Res. 2014; 42:D764–70. doi:10.1093/nar/gkt1168
5. Xu J, Li Y. Discovering disease-genes by topological features in human protein-protein interaction network. Bioinformatics. 2006; 22:2800–2805. doi:10.1093/bioinformatics/btl467
6. Brown G, Hem V, Katz K, Ovetsky M, Wallin C, Ermolaeva O, et al. Gene: a gene-centered information resource at NCBI. Nucleic Acids Res. 2014; 43(Database issue):D36–42. doi:10.1093/nar/gku1055
7. Pruitt KD, Brown GR, Hiatt SM, Thibaud-Nissen F, Astashyn A, Ermolaeva O, et al. RefSeq: an update on mammalian reference sequences. Nucleic Acids Res. 2014; 42(Database issue):D756–63. doi:10.1093/nar/gkt1114
8. The UniProt Consortium. Activities at the Universal Protein Resource (UniProt). Nucleic Acids Res. 2014; (Database issue)42:D191–8. doi:10.1093/nar/gkt1140

SYMBOL	HG19 LOCATION	OMIM	GENBANK	REFSEQ	UNIPROT
**AUTOSOMAL DOMINANT / RECESSIVE**					
BEST1	chr11:61717356 (+)	607854	7439	NM_004183	BEST1_HUMAN, O76090
NR2E3	chr15:72102894 (+)	604485	10002	NM_014249	NR2E3_HUMAN, Q9Y5X4
NRL	chr14:24549316 (-)	162080	4901	NM_006177	NRL_HUMAN, P54845
RHO	chr3:129247482 (+)	180380	6010	NM_001664	OPSD_HUMAN, P08100
RP1	chr8:55528627 (+)	603937	6101	NM_001618	RP1_HUMAN, P56715
RPE65	chr1:68894507 (-)	180069	6121	NM_000329	RPE65_HUMAN, Q16518
**AUTOSOMAL DOMINANT**					
CA4	chr17:58227302 (+)	114760	762	NM_000350	CAH4_HUMAN, P22748
CRX	chr19:48325099 (+)	602225	1406	NM_000554	CRX_HUMAN, O43186
FSCN2	chr17:79495417 (+)	607643	25794	NM_012418	FSCN2_HUMAN, O14926
GUCA1B	chr6:42151022 (-)	602275	2979	NM_002098	GUC1B_HUMAN, Q9UMX6
HK1	chr10:71029740 (+)	142600	3098	NM_033496	HXK1_HUMAN, P19367
IMPDH1	chr7:128032331 (-)	146690	3614	NM_001142573	IMDH1_HUMAN, P20839
KLHL7	chr7:23145353 (+)	611119	55975	NM_001031710	KLHL7_HUMAN, Q8IXQ5
OR2W3	chr1:248058889 (+)	—	343171	NM_001001957	OR2W3_HUMAN, Q7Z3T1
OTX2	chr14:57267425 (-)	600037	5015	NM_021728	OTX2_HUMAN, P32243
PRPF3	chr1:150293928 (+)	607301	9129	NM_004698	PRPF3_HUMAN, O43395
PRPF4	chr9:116037914 (+)	607795	9128	NM_003913	PRP4_HUMAN, O43172
PRPF6	chr20:62612431 (+)	613979	24148	NM_012469	PRP6_HUMAN, O94906
PRPF8	chr17:1553923 (-)	607300	10594	NM_006445	PRP8_HUMAN, Q6P2Q9
PRPF31	chr19:54618790 (+)	606419	26121	NM_015629	PRP31_HUMAN, Q8WWY3
PRPH2	chr6:42664333 (-)	179605	5961	NM_000322	PRPH2_HUMAN, P23942
RDH12	chr14:68168603 (+)	608830	145226	NM_152443	RDH12_HUMAN, Q96NR8
ROM1	chr11:62380213 (+)	180721	6094	NM_000327	ROM1_HUMAN, Q03395
RP9	chr7:33134410 (-)	607331	6100	NM_203288	RP9_HUMAN, Q8TA86
SEMA4A	chr1:156123322 (+)	607292	64218	NM_022367	SEM4A_HUMAN, Q9H3S1
SNRNP200	chr2:96940074 (-)	601664	23020	NM_014014	U520_HUMAN, O75643
TOPORS	chr9:32540542 (-)	609507	10210	NM_005802	TOPRS_HUMAN, Q9NS56

(***table continued on next page***)

S1 Table. Summary of the 110 genes related to RP/LCA phenotype inheritance (continued).

SYMBOL	HG19 LOCATION	OMIM	GENBANK	REFSEQ	UNIPROT
**AUTOSOMAL RECESSIVE**					
ABCA4	chr1:94458394 (-)	601691	24	NM_000350	ABCA4_HUMAN, P78363
ABHD12	chr20:25275379 (-)	613599	26090	NM_001042472	ABD12_HUMAN, Q8N2K0
AHI1	chr6:135604670 (-)	608894	54806	NM_017651	AHI1_HUMAN, Q8N157
AIPL1	chr17:6327057 (-)	604392	23746	NM_014336	AIPL1_HUMAN, Q9NZN9
ARL2BP	chr16:57279038 (+)	615407	23568	NM_012106	AR2BP_HUMAN, Q9Y2Y0
ARL6	chr3:97483365 (+)	608845	84100	NM_006407	ARL6_HUMAN, Q9H0F7
BBS1	chr11:66278077 (+)	209901	582	NM_024649	BBS1_HUMAN, Q8NFJ9
BBS2	chr16:56504301 (-)	606151	583	NM_031885	BBS2_HUMAN, Q9BXC9
C2orf71	chr2:29284556 (-)	613425	388939	NM_001029883	CB071_HUMAN, A6NGG8
C8orf37	chr8:96257141 (-)	614477	157657	NM_177965	CH037_HUMAN, Q96NL8
CABP4	chr11:67219886 (+)	608965	57010	NM_145200	CABP4_HUMAN, P57796
CC2D2A	chr4:15471489 (+)	612013	57545	NM_001080522	C2D2A_HUMAN, Q9P2K1
CEP290	chr12:88442790 (-)	610142	80184	NM_025114	CE290_HUMAN, O15078
CERKL	chr2:182401401 (-)	608381	375298	NM_001030311	CERKL_HUMAN, Q49MI3
CLRN1	chr3:150644354 (-)	606397	7401	NM_052995	CLRN1_HUMAN, P58418
CNGA1	chr4:47937994 (-)	123825	1259	NM_000087	CNGA1_HUMAN, P29973
CNGB1	chr16:57916244 (-)	600724	1258	NM_001297	CNGB1_HUMAN, Q14028
CRB1	chr1:197237334 (+)	604210	23418	NM_201253	CRUM1_HUMAN, P82279
CYP4V2	chr4:187112565 (+)	608614	285440	NM_207352	CP4V2_HUMAN, Q6ZWL3
DHDDS	chr1:26758773 (+)	608172	79947	NM_024887	DHDDS_HUMAN, Q86SQ9
DHX38	chr16:72127615 (+)	605584	9785	NM_014003	PRP16_HUMAN, Q92620
DTHD1	chr4:36283237 (+)	—	401124	NM_001136536	DTHD1_HUMAN, Q6ZMT9
EMC1	chr1:19542158 (-)	—	23065	NM_015047	EMC1_HUMAN, Q8N76
EYS	chr6:64429876 (-)	612424	346007	NM_001142800	EYS_HUMAN, Q5T1H1
FAM161A	chr2:62051983 (-)	613596	84140	NM_001201543	F161A_HUMAN, Q3B820
FLVCR1	chr1:213031597 (+)	609144	28982	NM_014053	FLVC1_HUMAN, Q9Y5Y0
GDF6	chr8:97154558 (-)	601147	392255	NM_001001557	GDF6_HUMAN, Q6KF10
GNPTG	chr16:1401900 (+)	607838	84572	NM_032520	GNPTG_HUMAN, Q9UJJ9
GPR125	chr4:22388997 (-)	612303	166647	NM_145290	GP125_HUMAN, Q8IWK6
GUCY2D	chr17:7905988 (+)	600179	3000	NM_000180	GUC2D_HUMAN, Q02846
HGSNAT	chr8:42995592 (+)	610453	138050	NM_152419	HGNAT_HUMAN, Q68CP4
IDH3B	chr20:2639041 (-)	604526	3420	NM_174855	IDH3B_HUMAN, O43837
IFT172	chr2:27667240 (-)	607386	26160	NM_015662	IF172_HUMAN, Q9UG01
IMPG2	chr3:100941390 (-)	607056	50939	NM_016247	IMPG2_HUMAN, Q9BZV3
INPP5E	chr9:139323067 (-)	613037	56623	NM_019892	INP5E_HUMAN, Q9NRR6
INVS	chr9:102861467 (+)	243305	27130	NM_183245	INVS_HUMAN, Q9Y283
IQCB1	chr3:121488608 (-)	609237	9657	NM_001023571	IQCB1_HUMAN, Q15051
KCNJ13	chr2:233630512 (-)	603208	3769	NM_001172417	IRK13_HUMAN, O60928
KIAA1549	chr7:138516127 (-)	613344	57670	NM_020910	K1549_HUMAN, Q9HCM3
KIZ	chr20:21106624 (+)	615780	15865	NM_001163022.1	KIZ_HUMAN, Q2M2Z5
LCA5	chr6:80194708 (-)	611408	167691	NM_152505	LCA5_HUMAN, Q86VQ0
LRAT	chr4:155665163 (+)	604863	9227	NM_004744	LRAT_HUMAN, O95237
MAK	chr6:10762956 (-)	154235	4117	NM_005906	MAK_HUMAN, P20794
MERTK	chr2:112656191 (+)	604705	10461	NM_006343	MERTK_HUMAN, Q12866
MVK	chr12:110011500 (+)	251170	4598	NM_000431	KIME_HUMAN, Q03426

(***table continued on next page***)

S1 Table. Summary of the 110 genes related to RP/LCA phenotype inheritance (continued).

SYMBOL	HG19 LOCATION	OMIM	GENBANK	REFSEQ	UNIPROT
**AUTOSOMAL RECESSIVE (continued)**					
NEK2	chr1:211836114 (-)	604043	4751	NM_001204183	NEK2_HUMAN, P51955
NEUROD1	chr2:182540833 (-)	601724	4760	NM_002500	NDF1_HUMAN, Q13562
NMNAT1	chr1:10003486 (+)	608700	64802	NM_022787	NMNA1_HUMAN, Q9HAN9
NPHP1	chr2:110880913 (-)	607100	4867	NM_207181	NPHP1_HUMAN, O15259
NPHP3	chr3:132399453 (-)	608002	27031	NM_153240	NPHP3_HUMAN, Q7Z494
NPHP4	chr1:5922870 (-)	607215	261734	NM_015102	NPHP4_HUMAN, O75161
PANK2	chr20:3869486 (+)	606157	80025	NM_153638	PANK2_HUMAN, Q9BZ23
PDE6A	chr5:149237519 (-)	180071	5145	NM_000440	PDE6A_HUMAN, P16499
PDE6B	chr4:619363 (+)	180072	5158	NM_000283	PDE6B_HUMAN, P35913
PDE6G	chr17:79617489 (-)	180073	5148	NM_002602	CNRG_HUMAN, P18545
PEX1	chr7:92116337 (-)	602136	5189	NM_001641	PEX1_HUMAN, O43933
PEX2	chr8:77892494 (-)	170993	5828	NM_000318	PEX2_HUMAN, P28328
PEX7	chr6:137143702 (+)	601757	5191	NM_000288	PEX7_HUMAN, O00628
PHYH	chr10:13319796 (-)	602026	5264	NM_006214	PAHX_HUMAN, O14832
PRCD	chr17:74523668 (+)	610598	768206	NM_001077620	PRCD_HUMAN, Q00LT1
PROM1	chr4:15969849 (-)	604365	8842	NM_006017	PROM1_HUMAN, O43490
RBP3	chr10:48381487 (-)	180290	5949	NM_002900	RET3_HUMAN, P10745
RD3	chr1:211649864 (-)	180040	343035	NM_000796	RD3_HUMAN, Q7Z3Z2
RDH11	chr14:68143517 (-)	607849	51109	NM_016026	RDH11_HUMAN, Q8TC12
RGR	chr10:86004809 (+)	600342	5995	NM_001012722	RGR_HUMAN, P47804
RLBP1	chr15:89753098 (-)	180090	6017	NM_000326	RLBP1_HUMAN, P12271
RP1L1	chr8:10463859 (-)	608581	94137	NM_178857	RP1L1_HUMAN, Q8IWN7
RPGRIP1	chr14:21756136 (+)	605446	57096	NM_015272	RPGR1_HUMAN, Q96KN7
SAG	chr2:234216309 (+)	181031	6295	NM_000541	ARRS_HUMAN, P10523
SDCCAG8	chr1:243419307 (+)	613524	10806	NM_006642	SDCG8_HUMAN, Q86SQ7
SLC7A14	chr3:170177342 (-)	615720	57709	NM_020949	S7A14_HUMAN, Q8TBB6
SPATA7	chr14:88851742 (+)	609868	55812	NM_018418	SPAT7_HUMAN, Q9P0W8
TTC8	chr14:89290918 (+)	608132	123016	NM_198309	TTC8_HUMAN, Q8TAM2
TTPA	chr8:63972047 (-)	600415	7274	NM_000370	TTPA_HUMAN, P49638
TUB	chr11:8040742 (+)	601197	7275	NM_003320	TUB_HUMAN, P50607
TULP1	chr6:35465651 (-)	602280	7287	NM_003322	TULP1_HUMAN, O00294
USH2A	chr1:215796236 (-)	608400	7399	NM_007123	USH2A_HUMAN, O75445
ZNF408	chr11:46722317 (+)	—	79797	NM_024741	ZN408_HUMAN, Q9H9D4
ZNF513	chr2:27600098 (-)	613598	130557	NM_144631	ZN513_HUMAN, Q8N8E2
**X-LINKED**					
OFD1	chrX:13752832 (+)	300170	8481	NM_003611	OFD1_HUMAN, O75665
PRPS1	chrX:106871654 (+)	311850	5631	NM_001204402	PRPS1_HUMAN, P60891
RP2	chrX:46696347 (+)	300757	6102	NM_001321	XRP2_HUMAN, O75695
RPGR	chrX:38143702 (-)	312610	6103	NM_001034853	RPGR_HUMAN, Q92834
