# Supplementary material for: Distilling a Visual Network of Retinitis Pigmentosa Gene-Protein Interactions to Uncover New Disease Candidates
Source: PLoS One. 2015 Aug 12;10(8):e0135307. doi: 10.1371/journal.pone.0135307 (PMC4534355; doi:10.1371/journal.pone.0135307)
Supplement: S2 Table — Comparison of the number of nodes (gene/protein) and edges (interactions) provided by the different datasets considered, as well as four intersections: joining interactions filtered from literature abstracts and full text (“Sparser ALL”); its combination with iHOP curated interactions (“Sparser + iHOP”); the merge of high-throughput data (“BioGRID + STRING”); and the result of processing all together (“ALL DBs”). “Hsap Nodes” were filtered from BioGRID human-specific interactions. “Unique Nodes/Edges” correspond to the simplified set of interactions that define the integrated graph from which the RP/LCA network was filtered out. “Unique Nodes” also include the RP/LCA genes that had no reported interactions on each dataset. Last three columns summarize the statistics for the networks computed over three sets of driver genes: non-syndromic, syndromic, or both (all). The latter constitutes the core of the RPGeNet webapp. Graph statistics are described in depth on [51]. (PDF) [file pone.0135307.s005.pdf]

S2 Table. Basic graph statistics for the RP genes interaction networks.

Comparison of the number of nodes (gene/protein) and edges (interactions) provided by the different datasets considered, as well as four intersections: joining interactions filtered from literature abstracts and full text ("Sparser ALL"); its combination with iHOP curated interactions ("Sparser + iHOP"); the merge of high-throughput data ("BioGRID + STRING"); and the result of processing all together ("ALL DBs"). "*Hsap* Nodes" were filtered from BioGRID human-specific interactions. "Unique Nodes/Edges" correspond to the simplified set of interactions that define the integrated graph from which the RP network was filtered out. "Unique Nodes" also include the RP genes that had no reported interactions on each dataset. Last three columns show results on RPGeNet interaction network, considering non-syndromic genes (75 RP/LCA genes) and syndromic genes (35 genes) separately and all together (110 genes). This last column data were used to feed the RPGeNet webapp. Graph statistics are described in depth on Newman *et al* [1]. Calculations for all the other columns are based on a preliminary set of 62 selected non-syndromic RP genes.

SET	Sparser Abstracts	Sparser Full-Text	Sparser All	iHOP	Sparser + iHOP	BioGRID	STRING	BioGRID + STRING	ALL DBs	RPGeNet v1.0 Genes Non-Syndr.	RPGeNet v1.0 Genes Syndromic	RPGeNet v1.0 Genes Total RP/LCA
**DATABASES**												
**Total Nodes**	68	380	388	136	488	51,877	15,993	63,068	63,139	63,139	63,139	63,139
***Hsap* Nodes**						*15,481*		*54,824*	*54,885*	*54,885*	*54,885*	*54,885*
**Total Edges**	64	628	692	141	833	761,693	926,130	1,687,823	1,688,656	1,688,656	1,688,656	1,688,656
**Unique Nodes**	120	413	421	157	500	15,394	15,999	26,889	26,962	22,371	22,368	22,372
**Unique Edges**	60	440	459	117	560	159,034	640,679	794,048	794,543	752,063	752,062	752.062
**RP_{FOUND}**	10 of 62	29 of 62	29 of 62	40 of 62	48 of 62	53 of 62	14 of 62	55 of 62	56 of 62	70 of 75	33 of 35	103 of 110
**SKELETON GRAPH**												
**#Nodes**	5	26	26	11	34	289	80	470	532	902	273	1,287
**#Edges**	4	33	33	8	43	647	150	1,148	1,277	3,563	781	5,883
**Density**	0.2000	0.0508	0.0508	0.0727	0.0383	0.0078	0.0237	0.0052	0.0045	0.0044	0.0105	0.0036
**#Islands**	1	1	1	3	2	1	1	1	1	1	1	1
**Global Cluster Coef.**	0	0.0845	0.0845	0	0.0957	0.0395	0.0244	0.0151	0.0176	0.0439	0.0434	0.0433
**Edge Connectivity**	0	0	0	0	0	0	0	0	0	0	0	0
**Graph Diameter**	1	6	6	2	6	12	11	14	14	11	8	10
**Graph Reciprocity**	0	0.1212	0.1212	0	0.1395	0.0433	0.1867	0.1132	0.1096	0.3441	0.3304	0.3678
**Avg. Graph Degree**	1.6000	2.5385	2.5385	1.4546	2.5294	4.4775	3.7500	4.8851	4.8008	7.9002	5.7216	9.1422
**Avg. Graph Closeness**	6.33E-2	2.10E-3	2.10E-3	1.04E-2	1.16E-3	1.04E-4	8.26E-4	3.22E-5	3.82E-5	1.34E-4	3.07E-4	8.47E-5
**Avg. Graph Betweenness**	0	10.7692	10.7692	0.2727	11.8824	998.0554	229.7625	1,778,062	2,043,927	2,890.857	842.319	3,998.789
**Avg. Local Cluster Coef.**	0	0.0931	0.0931	0	0.0918	0.0381	0.0049	0.0118	0.0179	0.0399	0.0449	0.0439
**Avg. Eigenvector Centrality**	0.7464	0.2722	0.2722	0.3879	0.2122	0.0642	0.1214	0.0619	0.0601	0.0462	0.07535	0.0362
**$RP_{CONNECTED}$**	5 of 62	15 of 62	15 of 62	11 of 62	20 of 62	46 of 62	14 of 62	49 of 62	56 of 62	70 of 75	33 of 35	103 of 110
**LEVEL 1 GRAPH**												
**#Nodes**	28	139	144	124	236	13,033	9,277	21,354	21,718	19,120	16,330	19,840
**#Edges**	28	174	181	108	274	54,202	38,887	155,903	163,420	262,113	110,536	327,409
**Density**	0.0370	0.0091	0.0088	0.0071	0.0049	0.0003	0.0005	0.0003	0.0003	0.0007	0.0004	0.0008
**#Islands**	5	12	12	24	22	3	1	3	1	1	1	1
**Global Cluster Coef.**	0.1017	0.0644	0.0627	0.0451	0.0512	0.0041	0.0016	0.0063	0.0067	0.0189	0.0046	0.0250
**Edge Connectivity**	0	0	0	0	0	0	1	0	0	0	0	0
**Graph Diameter**	3	7	7	4	11	7	7	7	9	7	6	7
**Graph Reciprocity**	0.2143	0.1839	0.1879	0.0556	0.1606	0.2069	1.0000	0.7086	0.7117	0.8401	0.8148	0.8447
**Avg. Graph Degree**	2.0000	2.5036	2.5139	1.7419	2.3220	8.3177	8.3835	14.6018	15.0493	27.4177	13.5378	33.0049
**Avg. Graph Closeness**	1.44E-3	5.96E-5	5.57E-5	6.66E-5	1.95E-5	4.43E-8	4.41E-5	5.08E-8	4.94E-8	5.84E-8	6.72E-8	5.40E-8
**Avg. Graph Betweenness**	1.3214	30.2158	32.1528	1.2661	40.0000	11,252.75	13,868.46	31,840.04	32,823.25	28,248.05	21,405.85	30,063.80
**Avg. Local Cluster Coef.**	0.1708	0.2811	0.2875	0.0432	0.2065	0.5709	0.0690	0.2619	0.2550	0.1768	0.2078	0.1655
**Avg. Eigenvector Centrality**	0.1887	0.0721	0.0709	0.0545	0.0470	0.0089	0.0101	0.0067	0.0067	0.0083	0.0077	0.0085
**$RP_{CONNECTED}$**	10 of 62	29 of 62	29 of 62	38 of 62	47 of 62	49 of 62	14 of 62	51 of 62	56 of 62	70 of 75	33 of 35	103 of 110

1. Newman MEJ, Barabási A-L, Watts DJ. The structure and dynamics of networks. 2006. p. 624
