# Supplementary material for: Distilling a Visual Network of Retinitis Pigmentosa Gene-Protein Interactions to Uncover New Disease Candidates
Source: PLoS One. 2015 Aug 12;10(8):e0135307. doi: 10.1371/journal.pone.0135307 (PMC4534355; doi:10.1371/journal.pone.0135307)
Supplement: S3 Table — Names of syndromic RP/LCA genes are marked in red, to distinguish from the non-syndromic ones. Conditional formatting on the cells in the spread sheet produce three horizontal barplots depicting the RP genes connectivity (“INDEGREE” for the incoming edges, “OUTDEGREE” for the outcoming, and “DEGREE” for the sum), based only on distinct interactions. Each barplot scale corresponds to five columns of the spread sheet, from levels zero (the skeleton graph) to 4 (the maximum graph level computed, yet webapp can only handle up to the level 2). Cells are filled in blue when the value on the left is smaller than the current cell value (therefore, the connectivity increases with the level). Note that for all RP/LCA genes connectivity saturates at level one; due to the methodology applied to build the skeleton network all their interactors are already included in that level. On the other hand, as expected, the total number of graph nodes and edges increases with the graph level (see bottom rows). RPF sums up the RP genes having at least one connection at each column. “Max” and “Avg” degree indicate the maximum and average degree, respectively; it is worth mentioning that the average indegree was 9.87 and the outdegree was 12.22 for the skeleton network. The red marks identify those RP genes that reach a connectivity degree greater than the maximum value of their average degree (59.58). Several genes are highlighted for both high indegree and outdegree connectivities; among those, one can mention: a centrosomal protein 290kDa (CEP290), a cone-rod homeobox protein (CRX), a DEAD-box protein probably involved in splicing (DHX38), the isocitrate dehydrogenase 3 beta subunit (IDH3B), several kinases (HK1, MAK, MERTK, and), several splicing splicing factors (PRPF3, PRPF4, PRPF6, PRPF8, PRPF31, and SNRNP200), rhodopsin (RHO), and an E3 ubiquitin-protein ligase (TOPORS). (PDF) [file pone.0135307.s006.pdf]

	GENE	INDEGREE					OUTDEGREE					DEGREE				
		LVL 0	LVL 1	LVL 2	LVL 3	LVL 4	LVL 0	LVL 1	LVL 2	LVL 3	LVL 4	LVL 0	LVL 1	LVL 2	LVL 3	LVL 4
1	ABCA4	4	7	7	7	7	4	6	6	6	6	8	13	13	13	13
2	ABHD12	0	0	0	0	0	3	3	3	3	3	3	3	3	3	3
3	AHI1	14	26	26	26	26	15	25	25	25	25	29	51	51	51	51
4	AIPL1	11	28	28	28	28	16	25	25	25	25	27	53	53	53	53
5	ARL2BP	4	9	9	9	9	6	12	12	12	12	10	21	21	21	21
6	ARL6	6	16	16	16	16	7	14	14	14	14	13	30	30	30	30
7	BBS1	10	15	15	15	15	4	4	4	4	4	14	19	19	19	19
8	BBS2	11	24	24	24	24	10	17	17	17	17	21	41	41	41	41
9	BEST1	2	3	3	3	3	6	9	9	9	9	8	12	12	12	12
10	C2ORF71	0	0	0	0	0	0	0	0	0	0	0	0	0	0	0
11	C8ORF37	0	0	0	0	0	1	1	1	1	1	1	1	1	1	1
12	CA4	5	6	6	6	6	4	6	6	6	6	9	12	12	12	12
13	CABP4	4	4	4	4	4	4	4	4	4	4	8	8	8	8	8
14	CC2D2A	5	13	13	13	13	4	13	13	13	13	9	26	26	26	26
15	CEP290	30	84	84	84	84	36	83	83	83	83	66	167	167	167	167
16	CERKL	10	19	19	19	19	15	18	18	18	18	25	37	37	37	37
17	CLRN1	1	1	1	1	1	0	1	1	1	1	1	2	2	2	2
18	CNGA1	6	7	7	7	7	6	8	8	8	8	12	15	15	15	15
19	CNGB1	17	23	23	23	23	18	25	25	25	25	35	48	48	48	48
20	CRB1	12	32	32	32	32	27	38	38	38	38	39	70	70	70	70
21	CRX	35	95	95	95	95	42	95	95	95	95	77	190	190	190	190
22	CYP4V2	3	5	5	5	5	4	5	5	5	5	7	10	10	10	10
23	DHDDS	2	7	7	7	7	5	8	8	8	8	7	15	15	15	15
24	DHX38	31	148	148	148	148	41	154	154	154	154	72	302	302	302	302
25	DTHD1	0	0	0	0	0	0	0	0	0	0	0	0	0	0	0
26	EMC1	0	0	0	0	0	7	8	8	8	8	7	8	8	8	8
27	EYS	2	3	3	3	3	3	3	3	3	3	5	6	6	6	6
28	FAM161A	1	1	1	1	1	1	1	1	1	1	2	2	2	2	2
29	FLVCR1	4	5	5	5	5	1	4	4	4	4	5	9	9	9	9
30	FSCN2	1	1	1	1	1	2	3	3	3	3	3	4	4	4	4
31	GDF6	6	7	7	7	7	6	7	7	7	7	12	14	14	14	14
32	GNPTG	13	23	23	23	23	14	25	25	25	25	27	48	48	48	48
33	GPR125	2	2	2	2	2	2	3	3	3	3	4	5	5	5	5
34	GUCA1B	6	8	8	8	8	8	10	10	10	10	14	18	18	18	18
35	GUCY2D	10	17	17	17	17	16	18	18	18	18	26	35	35	35	35
36	HGSNAT	0	0	0	0	0	0	0	0	0	0	0	0	0	0	0
37	HK1	18	41	41	41	41	26	51	51	51	51	44	92	92	92	92
38	IDH3B	13	33	33	33	33	17	40	40	40	40	30	73	73	73	73
39	IFT172	3	10	10	10	10	6	12	12	12	12	9	22	22	22	22
40	IMPDH1	5	7	7	7	7	9	14	14	14	14	14	21	21	21	21
41	IMPG2	0	0	0	0	0	0	0	0	0	0	0	0	0	0	0
42	INPP5E	9	11	11	11	11	11	12	12	12	12	20	23	23	23	23
43	INVS	16	22	22	22	22	12	16	16	16	16	28	38	38	38	38
44	IQCB1	34	187	187	187	187	9	10	10	10	10	43	197	197	197	197
45	KCNJ13	0	0	0	0	0	0	0	0	0	0	0	0	0	0	0
46	KIAA1549	6	8	8	8	8	5	7	7	7	7	11	15	15	15	15
47	KIZ	1	1	1	1	1	2	2	2	2	2	3	3	3	3	3
48	KLHL7	4	6	6	6	6	2	4	4	4	4	6	10	10	10	10
49	LCA5	4	4	4	4	4	4	4	4	4	4	8	8	8	8	8
50	LRAT	1	2	2	2	2	2	4	4	4	4	3	6	6	6	6
51	MAK	24	80	80	80	80	39	72	72	72	72	63	152	152	152	152
52	MERTK	23	55	55	55	55	28	56	56	56	56	51	111	111	111	111
53	MVK	9	16	16	16	16	13	19	19	19	19	22	35	35	35	35
54	NEK2	13	22	22	22	22	16	23	23	23	23	29	45	45	45	45
55	NEUROD1	24	60	60	60	60	25	66	66	66	66	49	126	126	126	126
56	NMNAT1	7	18	18	18	18	12	19	19	19	19	19	37	37	37	37
57	NPHP1	21	55	55	55	55	28	53	53	53	53	49	108	108	108	108
58	NPHP3	11	14	14	14	14	14	15	15	15	15	25	29	29	29	29
59	NPHP4	11	14	14	14	14	11	14	14	14	14	22	28	28	28	28
60	NR2E3	11	18	18	18	18	13	13	13	13	13	24	31	31	31	31
61	NRL	10	16	16	16	16	9	13	13	13	13	19	29	29	29	29
62	OFD1	30	66	66	66	66	33	70	70	70	70	63	136	136	136	136
63	OR2W3	14	272	272	272	272	22	272	272	272	272	36	544	544	544	544
64	OTX2	21	57	57	57	57	27	49	49	49	49	48	106	106	106	106
65	PANK2	9	21	21	21	21	14	17	17	17	17	23	38	38	38	38
66	PDE6A	4	6	6	6	6	5	8	8	8	8	9	14	14	14	14
67	PDE6B	5	7	7	7	7	9	14	14	14	14	14	21	21	21	21
68	PDE6G	4	4	4	4	4	2	2	2	2	2	6	6	6	6	6
69	PEX1	5	12	12	12	12	7	16	16	16	16	12	28	28	28	28
70	PEX2	3	5	5	5	5	3	3	3	3	3	6	8	8	8	8
71	PEX7	11	15	15	15	15	9	12	12	12	12	20	27	27	27	27
72	PHYH	5	15	15	15	15	10	16	16	16	16	15	31	31	31	31
73	PRCD	0	0	0	0	0	0	0	0	0	0	0	0	0	0	0
74	PROM1	8	21	21	21	21	14	22	22	22	22	22	43	43	43	43
75	PRPF3	25	111	111	111	111	29	122	122	122	122	54	233	233	233	233
76	PRPF31	39	183	183	183	183	46	191	191	191	191	85	374	374	374	374
77	PRPF4	37	196	196	196	196	38	195	195	195	195	75	391	391	391	391
78	PRPF6	38	241	241	241	241	47	254	254	254	254	85	495	495	495	495
79	PRPF8	12	42	42	42	42	32	85	85	85	85	44	127	127	127	127
80	PRPH2	1	1	1	1	1	1	2	2	2	2	2	3	3	3	3
81	PRPS1	15	35	35	35	35	24	41	41	41	41	39	76	76	76	76
82	RBP3	9	12	12	12	12	14	14	14	14	14	23	26	26	26	26
83	RD3	1	1	1	1	1	0	0	0	0	0	1	1	1	1	1
84	RDH11	4	7	7	7	7	8	11	11	11	11	12	18	18	18	18
85	RDH12	2	2	2	2	2	5	5	5	5	5	7	7	7	7	7
86	RGR	1	1	1	1	1	1	1	1	1	1	2	2	2	2	2
87	RHO	23	53	53	53	53	37	74	74	74	74	60	127	127	127	127
88	RLBP1	4	4	4	4	4	4	4	4	4	4	8	8	8	8	8
89	ROM1	0	1	1	1	1	3	4	4	4	4	3	5	5	5	5
90	RP1	11	19	19	19	19	16	19	19	19	19	27	38	38	38	38
91	RP1L1	1	1	1	1	1	0	0	0	0	0	1	1	1	1	1
92	RP2	4	4	4	4	4	5	5	5	5	5	9	9	9	9	9
93	RP9	3	4	4	4	4	4	8	8	8	8	7	12	12	12	12
94	RPE65	2	3	3	3	3	3	8	8	8	8	5	11	11	11	11
95	RPGR	17	42	42	42	42	21	40	40	40	40	38	82	82	82	82
96	RPGRIP1	7	9	9	9	9	6	7	7	7	7	13	16	16	16	16
97	SAG	15	28	28	28	28	14	30	30	30	30	29	58	58	58	58
98	SDCCAG8	28	57	57	57	57	36	59	59	59	59	64	116	116	116	116
99	SEMA4A	1	1	1	1	1	2	2	2	2	2	3	3	3	3	3
100	SLC7A14	0	0	0	0	0	0	0	0	0	0	0	0	0	0	0
101	SNRNP200	35	233	233	233	233	51	281	281	281	281	86	514	514	514	514
102	SPATA7	1	1	1	1	1	0	0	0	0	0	1	1	1	1	1
103	TOPORS	21	33	33	33	33	16	26	26	26	26	37	59	59	59	59
104	TTC8	6	7	7	7	7	5	8	8	8	8	11	15	15	15	15
105	TTPA	1	1	1	1	1	1	1	1	1	1	2	2	2	2	2
106	TUB	6	6	6	6	6	9	11	11	11	11	15	17	17	17	17
107	TULP1	2	2	2	2	2	1	1	1	1	1	3	3	3	3	3
108	USH2A	20	37	37	37	37	31	43	43	43	43	51	80	80	80	80
109	ZNF408	21	38	38	38	38	21	29	29	29	29	42	67	67	67	67
110	ZNF513	8	12	12	12	12	7	9	9	9	9	15	21	21	21	21
	RP_F	99	100	100	100	100	99	100	100	100	100	103	103	103	103	103
	Max Degree	39	272	272	272	272	51	281	281	281	281	86	544	544	544	544
	Avg Degree	9.87	29.71	29.71	29.71	29.71	12.22	29.87	29.87	29.87	29.87	22.09	59.58	59.58	59.58	59.58
	Total NODES	1,282	16,752	19,829	19,871	19,873	1,282	19,045	21,970	21,999	22,002	1,286	19,833	22,808	22,841	22,843
	Total EDGES	5,883	327,409	768,555	769,479	769,484	5,883	327,409	768,555	769,479	769,484	5,883	327,409	768,555	769,479	769,484
	Avg Degree	4.59	19.54	38.76	38.72	38.72	4.59	17.19	34.98	34.98	34.97	4.57	16.51	33.70	33.69	33.69
