# Supplementary material for: Distilling a Visual Network of Retinitis Pigmentosa Gene-Protein Interactions to Uncover New Disease Candidates
Source: PLoS One. 2015 Aug 12;10(8):e0135307. doi: 10.1371/journal.pone.0135307 (PMC4534355; doi:10.1371/journal.pone.0135307)
Supplement: S4 Table — Number of interactions found when at least one of the partners is an RP/LCA gene. Names of syndromic RP/LCA genes are marked in red, to distinguish from the non-syndromic ones. Rows at the bottom (6) sum up the number of nodes and interactions. Number of nodes are indicated as follows: RPF for RP genes found, and RPM for those missing in the data set; RPC_skel for those connected to another RP gene via the shortest path used to build the skeleton graph (level 0); and finally, RPC_level1 for RP genes integrated into the graph level 1 (as “parents” or “children” of a non-RP gene from the skeleton graph). “Total RP interactions” summarizes all the interactions found on each data source, and “Total DB interactions” indicates the global number of interactions contained in those sources. In all columns, “Sparser” corresponds to the Stanford-parser syntactical analysis approach. “Sparser ALL” shows the number of interactions found when merging (but not directly adding) the two input datasets defined for “Sparser Abstracts” and “Sparser FullText”. The same applies to the combined approaches: “Sparser + iHOP”, “BIOGRID + STRING”, and “ALL DBs”. The latter contains the resulting interactions found for each RP gene when all the five sets were used as input of the graph distiller program. While the previous columns show results based on a preliminary analysis of 62 RP/LCA genes, last three columns—shaded in light green—show the numbers for the gene sets used as driver genes for the RPGeNet, split into non-syndromic RP/LCA genes, RP/LCA syndromic genes, and both lists used together. (PDF) [file pone.0135307.s007.pdf]

	SET<br>GENE	Sparser Abstracts	Sparser FullText	iHOP	BIOGRID	STRING	Sparser ALL	Sparser +iHOP	BIOGRID +STRING	ALL DBs	Non-Syndromic RP/LCA GENES	Syndromic RP/LCA GENES	RPGeNet v1.0	SET<br>GENE
1	ABCA4	0	0	0	1	12	0	0	12	12	8		8	ABCA4
2	ABHD12											3	3	ABHD12
3	AHI1											23	29	AHI1
4	AIPL1										27		27	AIPL1
5	ARL2BP	0	1	1	10	8	1	1	8	1	9		10	ARL2BP
6	ARL6											10	13	ARL6
7	BBS1											10	14	BBS1
8	BBS2											13	21	BBS2
9	BEST1	0	0	7	2	0	0	7	2	7	7		8	BEST1
10	C2ORF71	0	0	0	0	0	0	0	0	0	0		0	C2ORF71
11	C8ORF37	0	0	0	0	0	0	0	0	0	1		1	C8ORF37
12	CA4	0	0	2	5	8	0	2	8	2	9		9	CA4
13	CABP4										8		8	CABP4
14	CC2D2A											8	9	CC2D2A
15	CEP290											32	66	CEP290
16	CERKL	0	2	0	0	36	2	2	36	2	26	0	25	CERKL
17	CLRN1	0	1	1	0	0	1	1	0	1		1	1	CLRN1
18	CNGA1	0	1	1	1	0	1	1	1	1	10		12	CNGA1
19	CNGB1	0	2	3	3	0	2	3	3	3	33		35	CNGB1
20	CRB1	0	0	3	3	74	0	3	74	3	31		39	CRB1
21	CRX	5	22	16	22	0	22	16	22	16	62		77	CRX
22	CYP4V2										7		7	CYP4V2
23	DHDDS	0	0	0	9	10	0	0	10	10	8		7	DHDDS
24	DHX38										64		72	DHX38
25	DTHD1											0	0	DTHD1
26	EMC1	0	0	0	72	0	0	0	72	72	7		7	EMC1
27	EYS	0	0	0	0	0	0	0	0	0	5		5	EYS
28	FAM161A	0	0	0	2	0	0	0	2	2	2		2	FAM161A
29	FLVCR1											4	5	FLVCR1
30	FSCN2	1	6	1	0	4	6	1	4	1	3		3	FSCN2
31	GDF6											11	12	GDF6
32	GNPTG											11	27	GNPTG
33	GPR125	0	0	0	1	4	0	0	4	4	4		4	GPR125
34	GUCA1B	0	1	3	0	0	1	3	0	3	15		14	GUCA1B
35	GUCY2D										22		26	GUCY2D
36	HGSNAT											0	0	HGSNAT
37	HK1										44		44	HK1
38	IDH3B	0	0	0	11	66	0	0	66	66	33		30	IDH3B
39	IFT172											9	9	IFT172
40	IMPDH1	0	1	0	13	12	1	1	12	1	11		14	IMPDH1
41	IMPG2	0	0	0	0	0	0	0	0	0	0		0	IMPG2
42	INPP5E											11	20	INPP5E
43	INVS											22	28	INVS
44	IQCB1											22	43	IQCB1
45	KCNJ13										0		0	KCNJ13
46	KIAA1549	0	0	1	9	6	0	1	6	1	11		11	KIAA1549
47	KIZ										3		3	KIZ
48	KLHL7	1	3	0	14	0	3	3	14	3	6		6	KLHL7
49	LCA5										8		8	LCA5
50	LRAT	0	0	2	2	0	0	2	2	2	3		3	LRAT
51	MAK	0	0	2	14	186	0	2	186	2	52		63	MAK
52	MERTK	1	8	0	16	100	8	8	100	8	45		51	MERTK
53	MVK	0	0	1	11	0	0	1	11	1	19		22	MVK
54	NEK2	0	2	0	56	0	2	2	56	2	22		29	NEK2
55	NEUROD1											25	49	NEUROD1
56	NMNAT1										18		19	NMNAT1
57	NPHP1											26	49	NPHP1
58	NPHP3											18	25	NPHP3
59	NPHP4											12	22	NPHP4
60	NR2E3	0	4	4	30	0	4	4	30	4	21		24	NR2E3
61	NRL	6	33	7	6	0	33	7	6	7	16		19	NRL
62	OFD1	0	7	1	26	0	7	1	26	1		24	63	OFD1
63	OR2W3										33		36	OR2W3
64	OTX2											28	48	OTX2
65	PANK2											14	23	PANK2
66	PDE6A	0	0	1	1	12	0	1	12	1	9		9	PDE6A
67	PDE6B	0	3	4	2	0	3	4	2	4	15		14	PDE6B
68	PDE6G	0	0	0	8	0	0	0	8	8	6		6	PDE6G
69	PEX1											9	12	PEX1
70	PEX2											5	6	PEX2
71	PEX7											13	20	PEX7
72	PHYH											10	15	PHYH
73	PRCD	0	0	0	0	0	0	0	0	0	0		0	PRCD
74	PROM1	0	0	1	4	0	0	1	4	1	21		22	PROM1
75	PRPF3	4	4	6	69	0	4	6	69	6	47		54	PRPF3
76	PRPF31	0	29	3	135	0	29	3	135	3	66		85	PRPF31
77	PRPF4										68		75	PRPF4
78	PRPF6	0	0	6	119	0	0	6	119	6	68		85	PRPF6
79	PRPF8	1	9	0	180	0	9	9	180	9	43		44	PRPF8
80	PRPH2	0	0	3	0	0	0	3	0	3	2		2	PRPH2
81	PRPS1											17	39	PRPS1
82	RBP3	0	0	2	1	0	0	2	1	2	20		23	RBP3
83	RD3										1		1	RD3
84	RDH11											7	12	RDH11
85	RDH12	0	0	1	10	0	0	1	10	1	6		7	RDH12
86	RGR	0	0	1	2	0	0	1	2	1	2		2	RGR
87	RHO	6	86	10	22	0	86	10	22	10	52		60	RHO
88	RLBP1	0	0	2	2	0	0	2	2	2	8		8	RLBP1
89	ROM1	0	2	1	51	0	2	1	51	1	3		3	ROM1
90	RP1	0	0	0	6	0	0	0	6	6	23		27	RP1
91	RP1L1										1		1	RP1L1
92	RP2	0	0	2	8	0	0	2	8	2	9		9	RP2
93	RP9	0	3	6	9	0	3	6	9	6	8		7	RP9
94	RPE65	0	1	3	5	0	1	3	5	3	6		5	RPE65
95	RPGR	10	42	4	25	0	42	4	25	4	31		38	RPGR
96	RPGRIP1										12		13	RPGRIP1
97	SAG	0	5	2	4	0	5	2	4	2	24		29	SAG
98	SDCCAG8											30	64	SDCCAG8
99	SEMA4A	0	0	1	1	0	0	1	1	1	3		3	SEMA4A
100	SLC7A14										0		0	SLC7A14
101	SNRNP200	1	8	1	170	0	8	1	170	1	71		86	SNRNP200
102	SPATA7	0	0	0	1	0	0	0	1	1	1		1	SPATA7
103	TOPORS	0	0	8	81	0	0	8	81	8	32		37	TOPORS
104	TTC8	0	0	1	3	0	0	1	3	1		9	11	TTC8
105	TTPA											2	2	TTPA
106	TUB											11	15	TUB
107	TULP1	0	1	0	0	0	1	1	0	1	3		3	TULP1
108	USH2A	0	6	4	1	0	6	4	1	4		33	51	USH2A
109	ZNF408										38		42	ZNF408
110	ZNF513	0	3	0	5	0	3	3	5	3	15		15	ZNF513
	RP_F	10	29	40	51	14	29	48	53	57	70	33	103	RP_F
	RP_M	52	33	22	11	48	33	14	9	5	5	2	7	RP_M
	RP_{C_skel}	5	15	11	46	14	15	20	49	56	70	33	103	RP_{C_skel}
	RP_{C_level1}	10	29	38	49	14	29	47	51	56	70	33	103	RP_{C_level1}
	TOTAL RP INTERAC-TIONS	36	296	129	1,264	538	296	158	1,709	339	1,427	483	2,430	TOTAL RP INTERAC-TIONS
	TOTAL DB INTERAC-TIONS	64	628	141	761,694	926,130	692	692	1,687,824	1,688,657	1,688,657	1,688,657	1,688,657	TOTAL DB INTERAC-TIONS
	SET	Sparser Abstracts	Sparser FullText	iHOP	BIOGRID	STRING	Sparser ALL	Sparser +iHOP	BIOGRID +STRING	ALL DBs	Non-Syndromic RP/LCA GENES	Syndromic RP/LCA GENES	RPGeNet v1.0	SET
